# Therapeutic efficacy and safety of Xuebijing in traumatic brain injury: systematic review and meta-analysis

**DOI:** 10.3389/fphar.2025.1669352

**Published:** 2025-12-08

**Authors:** Yihan Sun, Jiaqi Li, Xinyue Cui, Meiyu Yu, Boying Xiao, Junyuan Lv, Ziqi Li, Zhijing Wei, Niaoyao He, Zhuoyu Li, Hao Wen

**Affiliations:** 1 Department of Clinical Medicine, China Medical University, Shenyang, Liaoning, China; 2 Department of Trauma Center, The First Hospital of China Medical University, Shenyang, Liaoning, China; 3 Department of General Surgery, The Affiliated Hospital of Zunyi Medical University, Zunyi, Guizhou, China

**Keywords:** traumatic brain injury, Xuebijing, meta-analysis, inflammation, traditional Chinese medicine

## Abstract

**Background:**

Traumatic brain injury (TBI) represents a major global health challenge. Several clinical studies have suggested that Xuebijing (XBJ)—a patented Chinese botanical drug preparation may confer neuroprotective and anti-inflammatory benefits in TBI. This systematic review and meta-analysis aimed to evaluate the therapeutic efficacy and safety of XBJ as an adjunctive treatment for TBI.

**Materials and methods:**

A comprehensive search was performed across nine English and Chinese databases for randomized controlled trials (RCTs) evaluating XBJ in TBI patients, with records screened up to February 2025. Two independent reviewers conducted study selection and data extraction. Pooled estimates were calculated using fixed- or random-effects models, expressed as standardized mean differences (SMDs) or risk ratios (RRs) with 95% confidence intervals (CIs). Trial sequential analysis (TSA) and the GRADE framework were used to assess evidence robustness and certainty. Subgroup and sensitivity analyses explored potential dose–response patterns and sources of heterogeneity.

**Results:**

A total of 33 RCTs involving 3,215 patients with TBI met the inclusion criteria. Pooled analysis demonstrated that XBJ significantly improved neurological function, yielding higher Glasgow Coma Scale (GCS) scores (SMD = 0.66, 95% CI 0.51–0.80) and Glasgow Outcome Scale (GOS) scores (SMD = 0.78, 95% CI 0.39–1.16), while reducing all-cause mortality (RR = 0.56, 95% CI 0.44–0.69). XBJ also markedly decreased systemic inflammatory biomarkers, including C-reactive protein (CRP; SMD = −1.34), tumour necrosis factor-alpha (TNF-α; SMD = −0.98), and interleukin-6 (IL-6; SMD = −0.98) (all p < 0.01). No significant increase in adverse drug events (ADEs) was observed (RR = 1.32, 95% CI 0.58–2.96). Subgroup analyses indicated a dose-dependent relationship, with cumulative doses >1,400 mL and twice-daily intravenous administration associated with greater neurological and anti-inflammatory benefits.

**Conclusion:**

XBJ appears to be an effective and safe adjunctive therapy for TBI, improving neurological outcomes and reducing inflammatory responses. Dose-dependent effects support the optimization of treatment protocols.

## 1 Introduction

Traumatic brain injury (TBI) represents a significant global health challenge, frequently resulting from road traffic accidents, falls, sports-related incidents, or penetrating trauma (Blennow et al., 2016). It affects approximately 69 million individuals annually and ranks among the leading causes of mortality and long-term disability worldwide (Dewan et al., 2018). According to the Global Burden of Disease study, TBI accounts for an estimated 8.1 million disability-adjusted life years, with a global disability rate of 111 per 100,000 population (GBD, 2019). Beyond its acute effects, TBI often leads to persistent cognitive, physical, and psychological impairments, imposing a considerable socioeconomic burden on patients, caregivers, and healthcare systems (Peterson et al., 2021).

The pathophysiology of TBI encompasses both primary and secondary injury mechanisms. While primary injury stems from the initial mechanical insult, secondary injury evolves over hours to days via complex cascades including excitotoxicity, oxidative stress, blood–brain barrier (BBB) disruption, and neuroinflammation (Chodobski et al., 2011; Ng and Lee, 2019; Liu et al., 2024). These processes involve activation of microglia and peripheral immune cells, release of pro-inflammatory cytokines and chemokines, and systemic immune dysregulation (Cheng et al., 2012; Ng and Lee, 2019). In cases of severe or penetrating injury, increased vulnerability to infection and sepsis further exacerbates neuroinflammation through pathogen-associated molecular patterns (PAMPs) and dysregulated host responses (Corrigan et al., 2016; Maas et al., 2022). Although inflammation is integral to early repair, sustained or excessive immune activation may aggravate neuronal damage and impair recovery (Wang et al., 2015; Galgano et al., 2017). Consequently, immunomodulatory interventions have garnered growing attention as potential therapeutic strategies.

Traditional Chinese medicine (TCM) offers a broad spectrum of bioactive metabolites with anti-inflammatory and immunomodulatory properties (Riaz et al., 2023). Xuebijing (XBJ), a commercially available, patent-protected, government-approved TCM consisting of equal proportions of *Carthamus tinctorius* L. [Asteraceae; *Carthami flos*], *Paeonia lactiflora* Pall. [Paeoniaceae; *Paeoniae radix rubra*], *Ligusticum striatum* DC. [Apiaceae; *Chuanxiong rhizoma*], *Salvia miltiorrhiza* Bunge [Lamiaceae; *Salviae miltiorrhizae radix et rhizoma*] and *Angelica sinensis* (Oliv.) Diels [Apiaceae; *Angelicae sinensis radix*]—all verified in the “World Flora Online” on 23 January 2025, is approved in China for the treatment of sepsis, systemic inflammatory response syndrome (SIRS), and multiple organ dysfunction syndrome (MODS) (Huang et al., 2011). *The major bioactive metabolites identified in XBJ include hydroxysafflor yellow A*, paeoniflorin, ferulic acid, salvianolic acid and danshensu. These metabolites have been shown to inhibit NF-κB activation, reduce pro-inflammatory cytokine release, modulate immune responses, attenuate coagulation disturbances, neutralize endotoxins, and reduce oxidative stress, making XBJ well-suited for acute care (Song et al., 2019; Xie et al., 2019; Zhu et al., 2022).

Previous evidence has suggested that XBJ confers neuroprotective and neuroreparative benefits in the context of brain injury (Yadong et al., 2022; Wang et al., 2023). However, most existing clinical studies on XBJ in TBI are limited by small sample sizes, single-centre designs, and inconsistent outcome reporting. Furthermore, inadequate randomization, unclear blinding procedures, and heterogeneity in intervention protocols have contributed to variable and inconclusive findings. These methodological limitations underscore the need for a systematic review and meta-analysis to comprehensively evaluate the available evidence, identify sources of heterogeneity, and provide a clearer understanding of XBJ’s therapeutic potential and safety profile in TBI management.

## 2 Materials and methods

### 2.1 Protocols and registration

This systematic review and meta-analysis was conducted in accordance with the Preferred Reporting Items for Systematic Reviews and Meta-Analyses (PRISMA) guidelines, The study protocol was prospectively registered in the International Prospective Register of Systematic Reviews (PROSPERO; registration number CRD42023463977).

### 2.2 Search strategy

A comprehensive literature search was conducted across both English and Chinese databases from their inception to February 2025. The databases searched included PubMed, Embase, Cochrane Library, EBSCO, and Web of Science, as well as China National Knowledge Infrastructure (CNKI), Chinese Biomedicine Literature Database (CBM), VIP Database, and Wanfang Data. A combination of Medical Subject Headings (MeSH) and free-text terms was employed to identify eligible studies based on titles, abstracts, and keywords. Search terms included “Craniocerebral Trauma”, “Brain Injuries, Traumatic”, “Brain Injuries”, “Coma, Post-Head Injury”, “Cranial Nerve Injuries”, “Head Injuries, Closed”, “Head Injuries, Penetrating”, “Intracranial Hemorrhage, Traumatic”, “Skull Fractures”, and “Xuebijing” or “XBJ”. The search strategy was adapted to the indexing systems and syntax requirements of each database to maximise sensitivity. In addition, reference lists of all included articles and relevant systematic reviews were manually screened to identify additional eligible studies. Detailed search strategies for each database are provided in Supplementary Table S1.

### 2.3 Inclusion and exclusion criteria

This systematic review and meta-analysis was structured according to the PICOS framework to ensure methodological transparency and reproducibility. Population (P): Patients with a confirmed diagnosis of TBI, regardless of age, sex, ethnicity, or nationality. Intervention (I): Administration of *XBJ* as an adjunctive therapy in addition to conventional standard medical care. Comparator (C): Standard treatment or placebo, provided that XBJ administration was the only interventional difference between groups, thereby allowing an unconfounded evaluation of its therapeutic effect. Outcomes (O): Primary outcomes included the Glasgow Coma Scale (GCS), Glasgow Outcome Scale (GOS), and mortality. Secondary outcomes comprised inflammatory biomarkers—including C-reactive protein (CRP), tumour necrosis factor-alpha (TNF-α), and interleukin-6 (IL-6)—as well as adverse drug events (ADEs). Study design (S): Only randomized controlled trials (RCTs) were eligible for inclusion.

Trials were included irrespective of control intervention type, provided that the study design met the above criteria. When multiple publications reported findings from the same patient cohort, only the most recent or most comprehensive version was included. Eligible studies were restricted to those published in English or Chinese. Exclusion criteria were as follows: (1) non-randomized, quasi-experimental, or observational study designs; (2) studies involving non-TBI populations; (3) absence of a control group or use of XBJ in both arms; (4) lack of extractable or relevant outcome data; (5) preclinical animal or *in vitro* experiments; (6) reviews, protocols, conference abstracts, or commentaries; and (7) studies not compliant with ethical standards or lacking informed consent documentation.

### 2.4 Literature screening and data extraction

Two reviewers (YHS and JQL) independently screened all studies, with duplicates removed using EndNote X9 followed by manual verification. Titles and abstracts were initially assessed to exclude irrelevant records, after which full-text articles were reviewed to determine final eligibility. Data were extracted using a standardized data collection form, capturing study characteristics (first author, publication year, country, study design, and sample size), intervention details (XBJ dosage, administration frequency, and treatment duration), outcome measures (GCS, GOS, mortality, inflammatory biomarkers, and ADEs), and methodological variables relevant to bias assessment (randomization methods and allocation concealment, etc.). When dosage was reported as a range, the lowest value was recorded to avoid overestimation. Any discrepancies were resolved by a third reviewer (ZYL) or through consensus discussion.

### 2.5 Risk of bias assessment

The risk of bias in all included studies was assessed in accordance with the Cochrane Handbook for Systematic Reviews of Interventions (version 6.4) (Sterne et al., 2019). Two independent reviewers (HL and JYL) appraised methodological quality using the RoB 2 tool, evaluating five domains: randomization process, deviations from intended interventions, missing outcome data, outcome measurement, and selection of the reported result. Each study was classified as having a low risk of bias, some concerns, or a high risk of bias. Discrepancies were resolved through discussion or, if necessary, by consultation with a third reviewer (HW). Risk-of-bias visualisation was conducted using the robvis tool, which generated traffic-light plots and summary bar charts.

### 2.6 Trial sequential analysis

Trial sequential analysis (TSA) was performed using TSA software to estimate the required information size (RIS) and evaluate the robustness of the cumulative evidence (Claire et al., 2020). The x-axis represented the cumulative sample size, while the y-axis denoted the Z-score. Significance thresholds were defined at two-sided type I error levels of 5% and 1%. Results were deemed conclusive if the Z-curve crossed both the conventional significance boundary and the TSA monitoring boundary, suggesting that further trials may be redundant.

### 2.7 Statistical analyses

All statistical analyses were conducted using R (version 4.3.2), employing the meta, metafor, and dmetar packages. For dichotomous outcomes, risk ratios (RRs) with 95% confidence intervals (CIs) were calculated, while standardized mean differences (SMDs) with corresponding 95% CIs were used for continuous outcomes. Between-study heterogeneity was assessed using the Cochrane Q test and quantified with the I^2^ statistic. Low heterogeneity was defined as a P value ≥0.05 and I^2^ < 50%, under which a fixed-effects model was applied; otherwise, a random-effects model was used. Sensitivity analyses were performed to identify potential outliers, and subgroup analyses were conducted when more than six studies were available for a given outcome. Publication bias was evaluated using Begg’s and Egger’s tests, visual inspection of funnel plots, and the trim-and-fill method.

### 2.8 GRADE evidence quality assessment

The certainty of evidence was assessed using the GRADE (Grading of Recommendations, Assessment, Development and Evaluations) approach (Brożek et al., 2009). Judgements were based on study design, risk of bias, inconsistency, indirectness and imprecision. Certainty was rated as high, moderate, low or very low. The clinical importance of each outcome was scored from 1 to 9, reflecting low, moderate or critical relevance. GRADEpro software was used to support the evaluation process.

## 3 Results

### 3.1 Literature search and study selection

**FIGURE 1:**
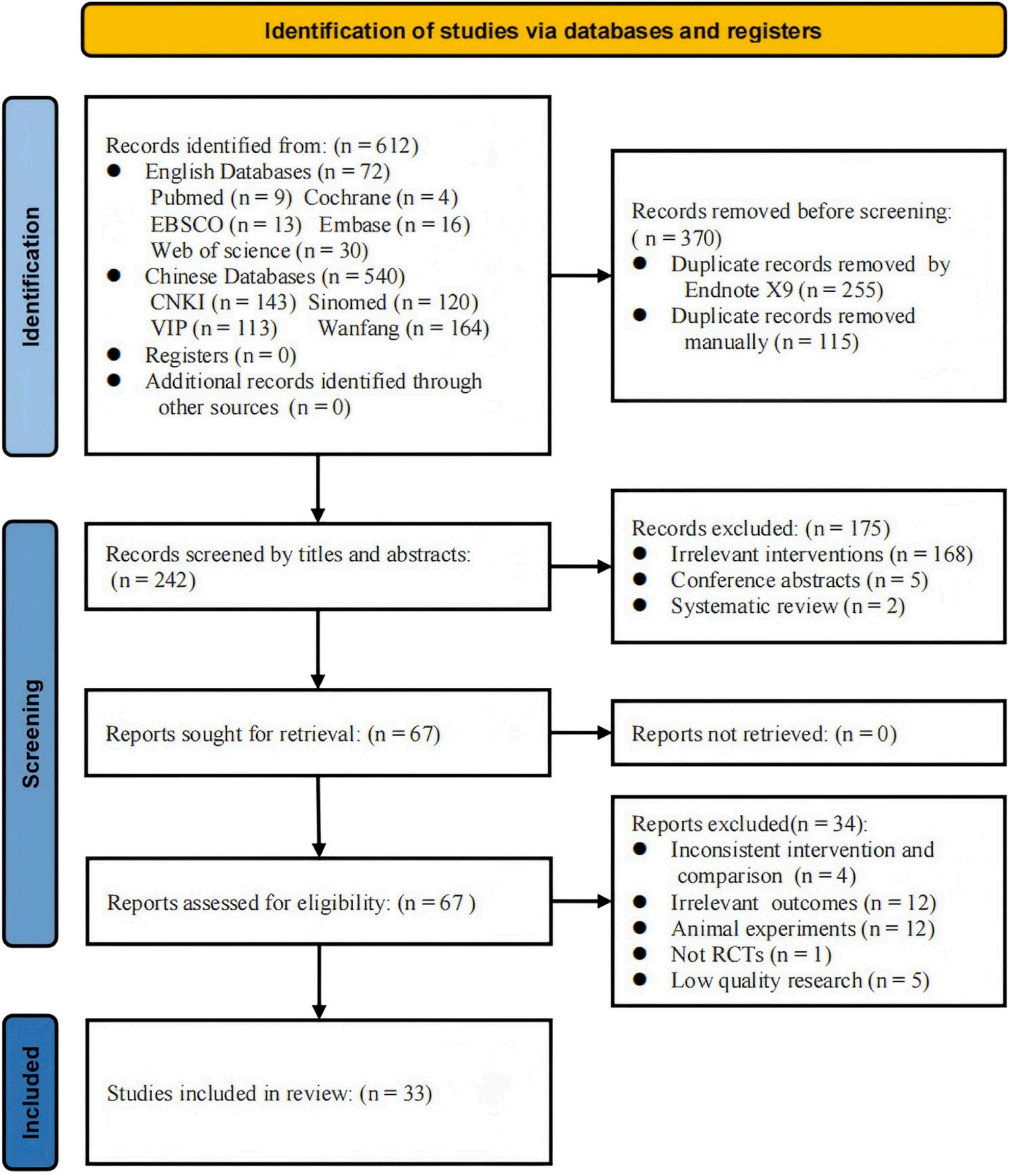
Flow diagram of the study selection process. The progression of the review process from the initial literature search to the ultimate meta-analysis. Each phase meticulously outlines the number of studies involved along with the rationale for study inclusion and exclusion.

A total of 612 records were retrieved from English (n = 72) and Chinese (n = 540) databases. After the removal of 370 duplicates, 242 records remained for title and abstract screening, during which 175 records were excluded due to irrelevant interventions, conference abstracts, or review articles. Subsequently, 67 full-text articles were assessed for eligibility, of which 34 were excluded for reasons including inconsistent interventions, non-relevant outcomes, use of animal models, non-randomized designs, or poor methodological quality. Ultimately, 33 studies (Lei et al., 2008; Zhang et al., 2008; Wang et al., 2010; Zhang et al., 2010; Yang et al., 2011; Chen et al., 2012; Diao et al., 2012; Li et al., 2012; Su and Xu, 2012; Pan et al., 2013; Ding et al., 2014; Shi and Qi, 2014; Wei et al., 2014; Ye and Yang, 2014; Zhao, 2014; Jiang et al., 2015; Zong, 2015; Li, 2016; Yu, 2016; Zhou et al., 2016; Chen et al., 2017; Dong et al., 2017; Li, 2018; Ma et al., 2018; Shui et al., 2018; Zhen et al., 2018; Zhou et al., 2018; Hu et al., 2019; Liu, 2020; Zhang et al., 2020; Gao, 2022; Yadong et al., 2022; Yin and Dong, 2024) met the inclusion criteria and were included in the final analysis (Figure 1).

### 3.2 Characteristics of included studies

The included trials were published between 2008 and 2024, with sample sizes ranging from 18 to 100 participants. The majority of studies were conducted in China and published in Chinese, with only one trial published in English (Yadong et al., 2022). Study populations comprised a balanced distribution of male and female participants, with mean ages ranging from 18 to 85 years. In all intervention arms, XBJ was administered alongside conventional therapy, whereas control groups received conventional therapy alone or in combination with other adjunctive treatments. XBJ was delivered at doses ranging from 20 to 300 mL, administered once to three times daily, for treatment durations of 5–21 days. Primary outcomes included the GCS, GOS, mortality, and inflammatory biomarkers. Additionally, several studies reported ADEs (Chen et al., 2017; Dong et al., 2017; Zhen et al., 2018; Hu et al., 2019) (Table 1).

**TABLE 1:** Basic characteristics of the included studies.

Author, year	Language	Design	Sample size		Gender, male		Age, years, mean ± SD*		Treatment		Details of XBJ			Outcomes
			C	T	C	T	C	T	C	T	Single dose, mL	Frequence, times/day	Duration, days	
Lei et al. (2008)	Chinese	RCT	30	31	16	17	46.4 ± 7.2	47.3 ± 7.2	CT	XBJ + CT	50	2	7	①
Zhang et al. (2008)	Chinese	RCT	20	20	32		40 ± 17.22	40 ± 17.22	CT	XBJ + CT	50	2	10	③
Wang et al. (2010)	Chinese	RCT	53	55	38	40	39.19 ± 11.33	38.81 ± 11.47	CT	XBJ + CT	50–100	1–2	14	①③④
Zhang et al. (2010)	Chinese	RCT	36	36	26	26	40.2 ± 11.3	38.8 ± 11.5	CT	XBJ + CT	50–100	1–2	14	①③④
Yang et al. (2011)	Chinese	RCT	18	20	23		22.5–65.7	22.5–65.7	CT	XBJ + CT	50	1	7	⑤⑥
Chen et al. (2012)	Chinese	RCT	28	28	35		38.4 ± 10.5	38.4 ± 10.5	CT	XBJ + CT	50–100	1–2	14	①③
Diao et al. (2012)	Chinese	RCT	42	43	54		38.1 ± 16.2	38.1 ± 16.2	CT	XBJ + CT	50	2	7	⑤⑥
Li et al. (2012)	Chinese	RCT	30	34	17	22	44.3	43.8	CT	XBJ + CT	50	2	21	⑤
Su and Xu (2012)	Chinese	RCT	30	30	32		40.0 ± 17.2	40.0 ± 17.2	CT	XBJ + CT	20	2	7	⑤⑥
Pan et al. (2013)	Chinese	RCT	36	36	45		39.72 ± 12.55	39.72 ± 12.55	CT	XBJ + CT	50	1	7	①④
Shi and Qi (2014)	Chinese	RCT	39	39	42		45.5 ± 2.8	45.5 ± 2.8	CT	XBJ + CT	100	1–2	14	③
Wei et al. (2014)	Chinese	RCT	30	30	—	—	18–70	18–70	CT	XBJ + CT	50	3	7	④
Ye and Yang (2014)	Chinese	RCT	30	30	18	16	35.8 ± 13.4	37.6 ± 15.1	CT	XBJ + CT	100	2	7	⑤
Zhao (2014)	Chinese	RCT	42	42	127		36.2 ± 10.4	36.2 ± 10.4	CT	XBJ + CT	50	2	7	①③⑤⑥
			42	42					CT + RO	XBJ + CT + RO				
Ding et al. (2014)	Chinese	RCT	25	25	39		43.5	43.5	CT	XBJ + CT	50	2	14	①④
Jiang et al. (2015)	Chinese	RCT	30	35	20	24	34.4 ± 8.4	35.1 ± 6.1	CT	XBJ + CT	30	2	10	①③⑤⑥
Zong (2015)	Chinese	RCT	39	39	25	23	44.2 ± 2.7	45.5 ± 2.8	CT	XBJ + CT	100	1	7	④
Li (2016)	Chinese	RCT	28	28	15	16	48.9 ± 1.8	48.2 ± 1.5	CT	XBJ + CT	300	1	7	④
Yu (2016)	Chinese	RCT	18	18	22		42.15 ± 6.43	42.15 ± 6.43	CT	XBJ + CT	50	2	7	①③④
Zhou et al. (2016)	Chinese	RCT	100	100	55	56	42.2 ± 3.5	43.7 ± 4.7	CT	XBJ + CT	50–100	1–2	14	⑤⑥
Chen et al. (2017)	Chinese	RCT	52	52	27	28	47.63 ± 10.87	48.12 ± 10.98	CT	XBJ + CT	20	2	14	①②④⑤⑥⑦
Dong et al. (2017)	Chinese	RCT	39	39	22	24	42.1 ± 4.5	41.8 ± 4.6	CT	XBJ + CT	100	2	7	⑦
Li (2018)	Chinese	RCT	50	50	29	27	35.5 ± 7.5	38.5 ± 8.5	CT	XBJ + CT	100	2	7	④
Ma et al. (2018)	Chinese	RCT	75	75	76		54.79 ± 6.32	54.79 ± 6.32	CT	XBJ + CT	50	2	7	⑤⑥
Shui et al. (2018)	Chinese	RCT	33	34	20	19	45.61 ± 10.82	46.57 ± 10.86	CT	XBJ + CT	30	2	10	⑤
Zhen et al. (2018)	Chinese	RCT	26	27	17	16	35.4 ± 11.4	36.5 ± 12.7	CT	XBJ + CT	60	2	14	④⑤⑥⑦
Zhou et al. (2018)	Chinese	RCT	31	31	22	19	36.3 ± 4.1	37.7 ± 4.5	CT + Gln	XBJ + CT + Gln	50	1	14	②③④⑤⑥
Hu et al. (2019)	Chinese	RCT	45	45	31	35	—	—	CT	XBJ + CT	50	2	14	③⑦
Liu (2020)	Chinese	RCT	30	30	17	19	36.2 ± 3.2	36.5 ± 3.4	CT + Gln	XBJ + CT + Gln	50	1	14	④⑤⑥
Zhang et al. (2020)	Chinese	RCT	50	46	49		48.79 ± 5.32	48.79 ± 5.32	CT	XBJ + CT	50	2	5	⑥
Gao (2022)	Chinese	RCT	60	60	32	34	44.36 ± 4.18	44.63 ± 4.75	CT + Gln	XBJ + CT + Gln	50	1	14	④⑤⑥
Yadong et al. (2022)	English	RCT	43	43	24	26	45.29 ± 6.25	43.88 ± 6.17	CT + MH	XBJ + CT + MH	50	3	5–7	②③⑤⑥
Yin and Dong (2024)	Chinese	RCT	34	34	18	19	50.12 ± 5.84	50.69 ± 6.28	CT + CSD	XBJ + CT + CSD	50	2	7	②

Abbreviations: RCT, randomized controlled trials; C, control group; T: treatment group; SD, standard deviation; XBJ, xuebijing; CT, conventional treatment; RO, rheum officinale; Gln, glutamine; MH, mild hypothermia; CSD, controlled stepwise decompression. ① Glasgow Coma Scale, GCS; ② Glasgow Outcome Scale, GOS; ③ mortality; ④ C- reactive protein, CRP; ⑤ tumor necrosis factor, TNF; ⑥ interleukin-6, IL-6; ⑦ adverse drug event, ADE;/, not mentioned. * Where available, age was extracted as mean ± SD; in the absence of such data, the reported range or mean was recorded to characterise the age distribution.

### 3.3 Risk of bias assessment

**FIGURE 2:**
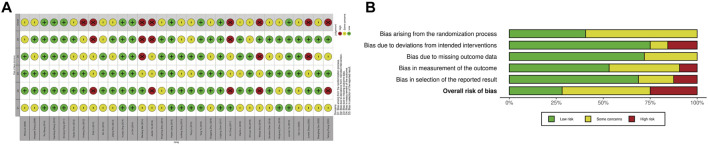
Risk of bias assessment. **(A)** Traffic light plots indicates the domain-level judgments for each individual study. **(B)** Weighted bar plots illustrates the distribution of risk-of-bias judgments within each bias domain.

Study quality was assessed using the ROB2 tool, as illustrated in Figure 2. All included trials reported randomization, though 19 studies [19,21,22,26,29,31-41,45,48,50] did not describe the method of sequence generation and were rated as unclear risk in this domain. Most studies showed low risk regarding adherence to intended interventions; however, five studies [19,27,29,32,45] were rated as high risk due to deviations that could affect outcomes, and three [20,21,40] were rated as unclear. For missing outcome data, nine studies [26-29,31-33,45,49] did not report handling procedures and were judged as unclear. Overall, nine studies [23-25,30,42-44,46,47] were classified as low risk, eight [19,26,27,29,32,33,35,45] as high risk, and the remainder as having some concerns.

### 3.4 Primary outcomes

#### 3.4.1 GCS

**FIGURE 3:**
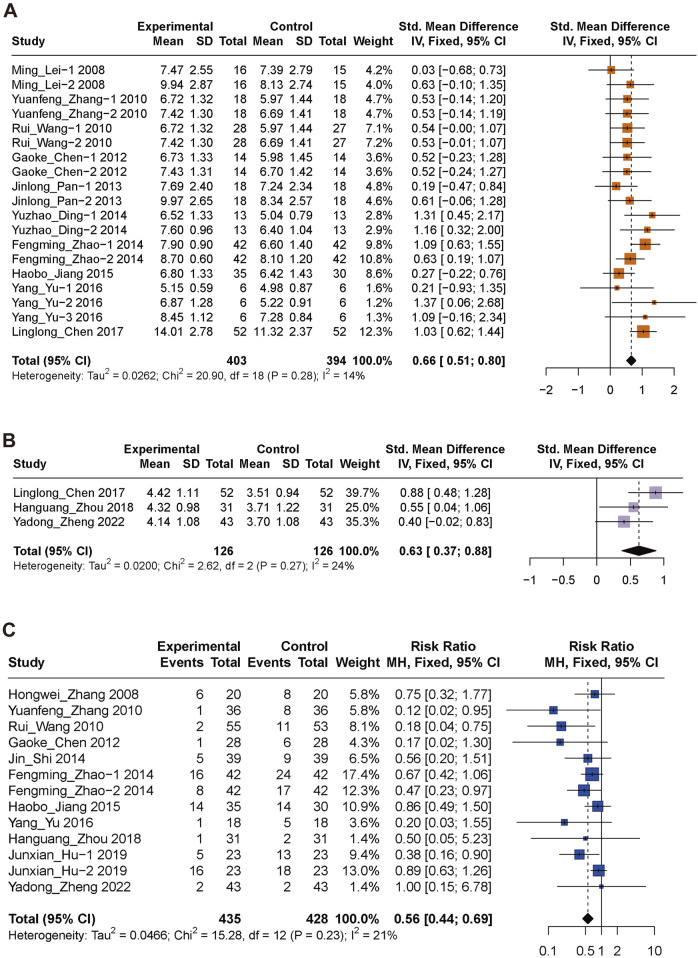
Forest plots for primary outcomes. Forest plots illustrates the effects of XBJ compared to the control group on **(A)** GCS, **(B)** GOS and **(C)** mortality in TBI. XBJ, Xuebijing; GCS, Glasgow Coma Scale; GOS, Glasgow Outcome Scale; TBI, traumatic brain injury.

Ten studies (Lei et al., 2008; Wang et al., 2010; Zhang et al., 2010; Chen et al., 2012; Pan et al., 2013; Ding et al., 2014; Zhao, 2014; Jiang et al., 2015; Yu, 2016; Chen et al., 2017), comprising 19 study arms and a total of 797 patients, reported GCS outcomes. A fixed-effects meta-analysis showed a significant improvement in GCS scores in the XBJ group compared with controls (SMD = 0.66; 95% CI: 0.51 to 0.80; Figure 3A). Statistical heterogeneity was low and not significant (P = 0.28; I^2^ = 14%), and sensitivity analysis was therefore not required.

#### 3.4.2 GOS

Four trials (Chen et al., 2017; Zhou et al., 2018; Yadong et al., 2022; Yin and Dong, 2024), involving 320 participants, reported GOS scores. A random-effects meta-analysis showed a significant improvement in the XBJ group compared with controls (SMD = 0.78; 95% CI: 0.39 to 1.16; Figure 3B), with substantial heterogeneity (I^2^ = 63%, P = 0.04). Leave-one-out sensitivity analysis identified one potential outlier [55] (Supplementary Figure S1). After its exclusion, the pooled effect remained significant (SMD = 0.63; 95% CI: 0.37–0.88), and heterogeneity was reduced to a non-significant level (I^2^ = 23.7%, P = 0.27; Supplementary Figure S1).

#### 3.4.3 Mortality rate

Eleven studies (Zhang et al., 2008; Wang et al., 2010; Zhang et al., 2010; Chen et al., 2012; Shi and Qi, 2014; Zhao, 2014; Jiang et al., 2015; Yu, 2016; Zhou et al., 2018; Hu et al., 2019; Yadong et al., 2022), involving 863 patients across 13 study arms, evaluated mortality. A fixed-effects meta-analysis showed a significantly lower mortality rate in the XBJ group compared with controls (RR = 0.56; 95% CI: 0.44 to 0.69; Figure 3C). Heterogeneity was low and not statistically significant (I^2^ = 21%, P = 0.23), and sensitivity analysis was therefore not required.

### 3.5 Secondary outcomes

#### 3.5.1 CRP

Fourteen studies (Wang et al., 2010; Zhang et al., 2010; Pan et al., 2013; Ding et al., 2014; Wei et al., 2014; Zong, 2015; Li, 2016; Yu, 2016; Chen et al., 2017; Li, 2018; Zhen et al., 2018; Zhou et al., 2018; Liu, 2020; Gao, 2022), comprising 26 study arms and 1,039 patients, assessed CRP levels. A random-effects meta-analysis showed that XBJ significantly reduced CRP compared with controls (SMD = −1.34; 95% CI: −1.82 to −0.85), although heterogeneity was substantial (I^2^ = 86%, P < 0.01; Supplementary Figure S2). Leave-one-out sensitivity analysis identified one potential outlier (Li, 2018). After its exclusion, the effect remained significant (SMD = −0.99; 95% CI: −1.28 to −0.69), with a modest reduction in heterogeneity (I^2^ = 77%, P < 0.01; Supplementary Figure S2), indicating that the observed variability was broadly distributed across studies and that the result was robust under the random-effects model.

#### 3.5.2 TNF-α

Sixteen studies (Yang et al., 2011; Diao et al., 2012; Li et al., 2012; Su and Xu, 2012; Ye and Yang, 2014; Zhao, 2014; Jiang et al., 2015; Zhou et al., 2016; Chen et al., 2017; Ma et al., 2018; Shui et al., 2018; Zhen et al., 2018; Zhou et al., 2018; Liu, 2020; Gao, 2022; Yadong et al., 2022), comprising 31 study arms and 1,453 patients, assessed TNF-α levels. A random-effects meta-analysis showed a significant reduction in the XBJ group compared with controls (SMD = −0.98; 95% CI: −1.30 to −0.67; Supplementary Figure S3), although heterogeneity was considerable (I^2^ = 83%, P < 0.01). Leave-one-out sensitivity analysis identified one outlier study (Li et al., 2012). After its exclusion, the pooled effect remained significant (SMD = −0.89; 95% CI: −1.14 to −0.65), and heterogeneity decreased to 72% (Supplementary Figure S3), indicating improved consistency while confirming the robustness of the finding under the random-effects model.

#### 3.5.3 IL-6

Fourteen studies (Yang et al., 2011; Diao et al., 2012; Su and Xu, 2012; Zhao, 2014; Jiang et al., 2015; Zhou et al., 2016; Chen et al., 2017; Ma et al., 2018; Zhen et al., 2018; Zhou et al., 2018; Liu, 2020; Zhang et al., 2020; Gao, 2022; Yadong et al., 2022), comprising 26 study arms and 1,355 patients, evaluated IL-6 levels. A random-effects meta-analysis showed a significant reduction in IL-6 levels in the XBJ group compared with controls (SMD = −0.98; 95% CI: −1.32 to −0.65; Supplementary Figure S4), although heterogeneity was substantial (I^2^ = 83%, P < 0.01). Leave-one-out sensitivity analysis identified one potential outlier (Zhang et al., 2020). After its exclusion, the effect remained statistically significant (SMD = −0.87; 95% CI: −1.10 to −0.64), and heterogeneity decreased to 67% (Supplementary Figure S4), supporting the robustness of the overall estimate despite residual variability.

#### 3.5.4 ADEs

ADEs were reported in four studies (Chen et al., 2017; Dong et al., 2017; Zhen et al., 2018; Hu et al., 2019) involving 325 patients. A fixed-effects meta-analysis showed no significant difference in the incidence of ADEs between the XBJ and control groups (RR = 1.32; 95% CI: 0.58 to 2.96; Supplementary Figure S5). Heterogeneity was low and not statistically significant (I^2^ = 14%, P = 0.31), indicating consistency across studies.

### 3.6 Subgroup analysis

Subgroup analyses demonstrated a dose-dependent effect of XBJ on neurological function, and inflammatory biomarkers (Table 2; Supplementary Figures S6-10). Progressive improvements in GCS scores were observed with increasing total dosage (≤350 mL: SMD = 0.37; ≤700 mL: SMD = 0.82; ≤1,400 mL: SMD = 1.14; P  for subgroup < 0.01), indicating enhanced neurological recovery with higher cumulative XBJ administration. Inflammatory markers also showed dose-related reductions. The most substantial decrease in TNF-α was observed in the >1,400 mL subgroup (P for subgroup < 0.01), while IL-6 reduction was also most pronounced in the same group (P for subgroup < 0.01), supporting a strong anti-inflammatory effect at higher doses. Moreover, twice-daily administration was associated with greater reductions in CRP (P for subgroup < 0.01). In addition, treatment durations up to 2 weeks yielded the most notable reductions in TNF-α (P for subgroup = 0.04) and IL-6 (P for subgroup = 0.05), suggesting that sustained exposure enhances immunomodulatory efficacy. However, a seemingly divergent trend was observed for mortality, with a lower risk of death in the ≤350 mL group (RR = 0.47) compared to the ≤700 mL group (RR = 0.78; P for subgroup = 0.03), suggesting that higher-dose groups may have included patients with more severe baseline conditions.

**TABLE 2:** Results for subgroup analyses.

Subgroup	GCS		Mortality		CRP		TNF-α		IL-6	
	SMD [95% CI]	P*	RR [95% CI]	P*	SMD [95% CI]	P*	SMD [95% CI]	P*	SMD [95% CI]	P*
**Single dose (mL)**		NA		1.00		0.37		0.02		**<0.01**
≤50	0.66 [0.51; 0.80]		0.56 [0.44; 0.70]		−1.05 [-1.42; -0.69]		−0.87 [-1.14; -0.59]		−0.93 [-1.27; -0.60]	
≤100	–		0.56 [0.20; 1.51]		−1.23 [-2.54; 0.08]		−2.87 [-4.57; -1.17]		−2.20 [-2.89; -1.51]	
>100	–		–		−1.56 [-2.16; -0.96]		–		–	
**Frequency *(times/day)***		0.07		0.05		**<0.01**		0.05		0.67
Once	0.50 [0.27; 0.72]		0.28 [0.14; 0.54]		−0.94 [-1.29; -0.59]		−0.69 [-1.03; -0.36]		−0.87 [-1.15; -0.60]	
Twice	0.77 [0.58; 0.95]		0.65 [0.51; 0.82]		−1.71 [-2.63; -0.79]		−1.13 [-1.58; -0.68]		−1.10 [-1.64; -0.55]	
Thrice	–		1.00 [0.15; 6.78]		0.07 [-0.44; 0.57]		−1.38 [-1.85; -0.91]		−1.06 [-1.52; -0.61]	
**Daily dosage (mL)**		0.15		0.18		0.99		0.17		0.46
<100	0.57 [0.36; 0.76]		0.42 [0.26; 0.69]		−1.12 [-1.55; -0.69]		−0.81 [-1.21; -0.41]		−0.89 [-1.17; -0.60]	
≥100	0.79 [0.56; 1.02]		0.61 [0.48; 0.79]		−1.12 [-1.80; -0.45]		−1.25 [-1.75; -0.76]		−1.21 [-2.03; -0.40]	
**Treatment duration (weeks)**		0.18		0.66		0.15		**0.04**		0.05
≤1	0.59 [0.42; 0.76]		0.52 [0.37; 0.74]		−0.97 [-1.50; -0.44]		−0.73 [-0.96; -0.50]		−0.85 [-1.24; -0.46]	
≤2	0.80 [0.55; 1.06]		0.58 [0.43; 0.78]		−1.45 [-1.87; -1.04]		−1.88 [-2.85; -0.91]		−1.47 [-1.97; -0.98]	
>2	–		–		–		−1.37 [-2.32; -0.42]		–	
**Total dosage (mL)**		**<0.01**		**0.03**		0.08		**<0.01**		**<0.01**
≤350	0.37 [0.15; 0.60]		0.47 [0.35; 0.63]		−0.65 [-1.07; -0.23]		−0.40 [-0.60; -0.20]		−0.64 [-0.93; -0.36]	
≤700	0.82 [0.63; 1.01]		0.78 [0.55; 1.11]		−1.30 [-1.73; -0.88]		−1.28 [-1.76; -0.81]		−1.28 [-1.96; -0.61]	
≤1,400	1.14 [0.44; 1.84]		–		−1.92 [-4.54; 0.70]		−1.47 [-2.08; -0.85]		−1.06 [-1.52; -0.61]	
>1,400	–		–		−1.33 [-1.77; 0.90]		−2.57 [-4.93; -0.22]		−2.20 [-2.89; -1.51]	
										

Abbreviations: RR, risk ratio; CI, confidence interval; SMD, standardized mean difference; GCS, glasgow coma scale; GOS, glasgow outcome scale; TBI, traumatic brain injury; CRP, C-reactive protein; TNF-α, tumor necrosis factor-alpha; IL-6, interleukin-6. P*, P-value for subgroup difference.

Bold values indicate statistically significant results (p < 0.05).

### 3.7 Publication bias

**FIGURE 4:**
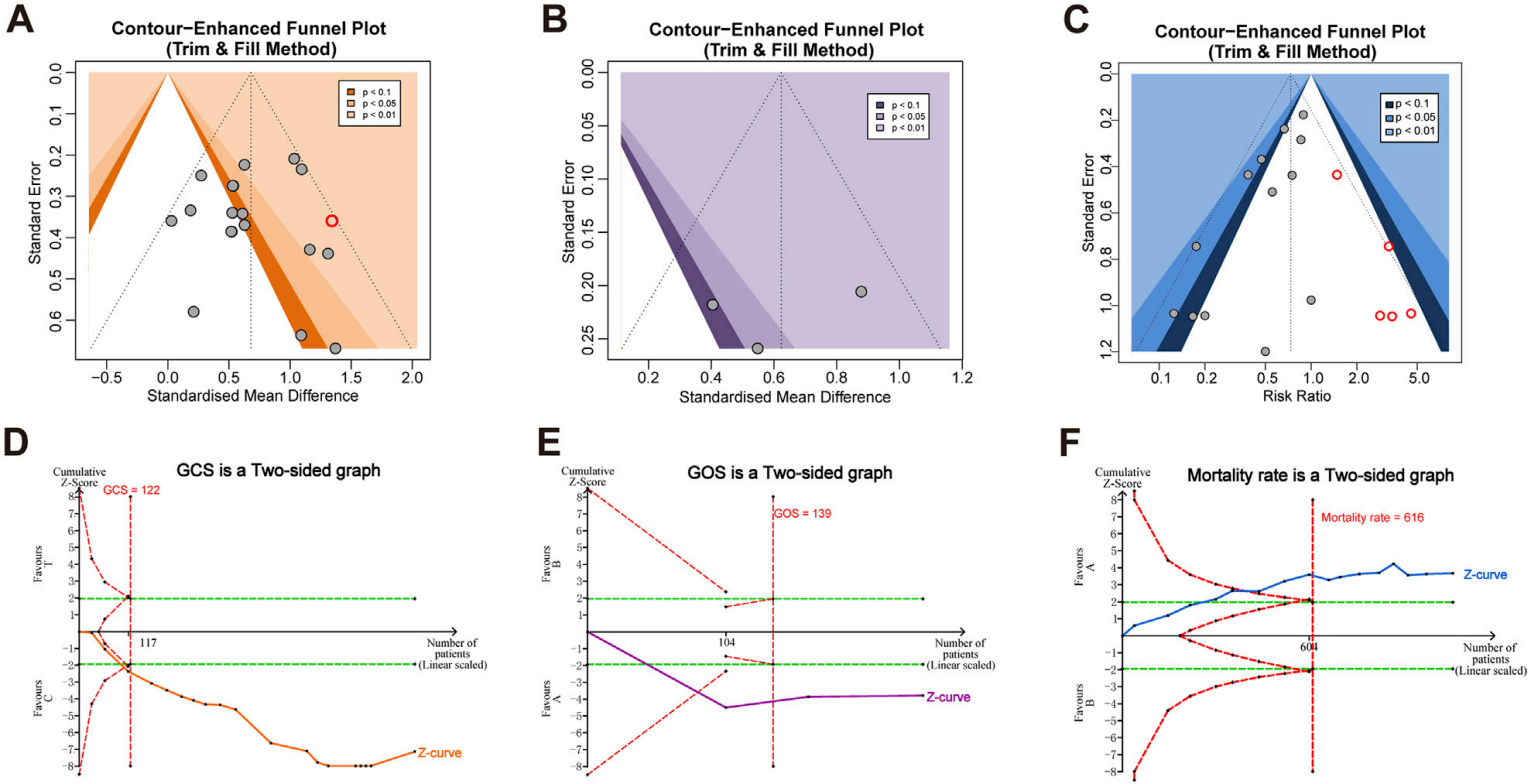
Publication bias and trial sequential analysis. Trim-and-fill funnel plots for **(A)** GCS, **(B)** GOS and **(C)** mortality. Trial sequential analysis for **(D)** GCS, **(E)** GOS and **(F)** mortality. GCS, Glasgow Coma Scale; GOS, Glasgow Outcome Scale.

Both Begg’s and Egger’s tests were employed to assess publication bias via funnel plot asymmetry across key outcomes. For GCS, neither Begg’s test (z = 1.08, P = 0.2781) nor Egger’s test (t = −0.12, P = 0.9053) indicated significant asymmetry, suggesting a low risk of publication bias. This was further supported by the trim-and-fill method, which imputed only one study without altering the overall effect size (Figure 4A). Similarly, for GOS, Begg’s test (z = 0.68, P = 0.4969) and Egger’s test (t = 0.57, P = 0.6962) showed no evidence of bias, and no studies were added through the trim-and-fill analysis (Figure 4B). In contrast, for mortality, Egger’s test indicated significant funnel plot asymmetry (t = −3.71, P = 0.0035), while Begg’s test showed a trend toward significance (z = −1.71, P = 0.0876). The trim-and-fill method imputed five potentially missing studies (Figure 4C), suggesting the presence of publication bias, possibly attributable to small-study effects.

### 3.8 TSA

In this analysis, TSA was applied to account for random errors, adjust for repeated significance testing, and assess whether the RIS had been reached to support firm conclusions. The cumulative Z-curves for GCS, GOS, and mortality each crossed both the conventional significance thresholds and the trial sequential monitoring boundaries (Figures 4D–F). The RIS was achieved for GCS and GOS, indicating conclusive evidence for these outcomes. Although the RIS for mortality was not fully attained, the Z-curve still crossed the efficacy boundary, suggesting that additional trials are unlikely to substantially alter the observed effect. Collectively, these results provide strengthened and statistically robust evidence supporting the clinical efficacy of XBJ in the treatment of TBI.

### 3.9 GRADE evidence quality assessment

The GRADE assessment of XBJ for TBI demonstrated variable levels of certainty across outcomes (Table 3). For GCS, evidence from ten RCTs was rated as high certainty, showing a significant improvement with XBJ (SMD = 0.66; 95% CI: 0.51–0.80). GOS, assessed in four RCTs, was supported by moderate-certainty evidence (SMD = 0.78; 95% CI: 0.39–1.16), downgraded due to risk of bias. The mortality outcome was supported by high-certainty evidence, with a pooled RR of 0.56 (95% CI: 0.44–0.69), corresponding to 141 fewer deaths per 1,000 patients. By contrast, the evidence supporting reductions in inflammatory biomarkers—CRP, TNF-α, and IL-6—was rated as very low certainty, primarily due to serious risk of bias and suspected publication bias. The respective effect sizes were SMD = −1.12 for CRP, −0.98 for TNF-α, and −0.98 for IL-6. Despite favorable trends, the low confidence in these findings highlights the need for further high-quality trials to validate the anti-inflammatory effects of XBJ.

**TABLE 3:** GRADE evidence quality assessment.

Certainty assessment							№ of patients		Effect		Certainty	Importance
№ of studies	Study design	Risk of bias	Inconsistency	Indirectness	Imprecision	Other considerations	Xuebijing	Control	Relative (95% CI)	Absolute (95% CI)		
Glasgow coma scale (follow-up: range 7 days to 14 days; assessed with: SMD)												
10	Randomized trials	Not serious	Not serious	Not serious	Not serious	Dose response gradient	403	394	-	SMD 0.66 SD higher (0.51 higher to 0.8 higher)	⨁⨁⨁⨁ High	CRITICAL
Glasgow outcome scale (follow-up: range 5 days to 14 days; assessed with: SMD)												
4	Randomized trials	Very serious^a^	Not serious	Not serious	Not serious	Dose response gradient	160	160	-	SMD 0.78 SD higher (0.39 higher to 1.16 higher)	⨁⨁⨁⃝ Moderate	CRITICAL
Mortality(follow-up: range 5 days to 14 days; assessed with: RR)												
11	Randomized trials	serious^a^	Not serious	Not serious	Not serious	Publication bias strongly suspected strong association Dose response gradient^b^	78/435 (17.9%)	137/428 (32.0%)	RR 0.56 (0.44–0.69)	141 fewer per 1,000 (from 179 fewer to 99 fewer)	⨁⨁⨁⨁ High	CRITICAL
C- reactive protein (follow-up: range 7 days to 14 days; assessed with: SMD)												
14	Randomized trials	serious^c^	Very serious^d^	Not serious	Not serious	Publication bias strongly suspected Dose response gradient^b^	520	519	-	SMD 1.12 SD lower (1.5 lower to 0.73 lower)	⨁⃝⃝⃝ Very low	IMPORTANT
Tumor necrosis factor-α (follow-up: range 5 days to 21 days; assessed with: SMD)												
16	Randomized trials	Very serious^a^ ^,^ ^e^	Very serious^d^	Not serious	Not serious	Publication bias strongly suspected Dose response gradient^b^	736	717	-	SMD 0.98 SD lower (1.3 lower to 0.67 lower)	⨁⃝⃝⃝ Very low	IMPORTANT
Interleukin-6 (follow-up: range 5 days to 14 days; assessed with: SMD)												
14	Randomized trials	Very serious^a^ ^,^ ^c^ ^,^ ^e^	Very serious^d^	Not serious	Not serious	Publication bias strongly suspected Dose response gradient^b^	682	673	-	SMD 0.98 SD lower (1.32 lower to 0.65 lower)	⨁⃝⃝⃝ Very low	IMPORTANT

Abbreviations: CI, confidence interval; RR, risk ratio; SMD, standardized mean difference. Explanations:

^a^ Inappropriate intervention measures.

^b^ Contour-Enhanced Funnel Plot (Trim & Fill Method) indicate a potential risk of publication bias.

^c^ Lack of blinding leads to detection bias.

^d^ P<0.01 and I^2^>75% exist simultaneously.

^e^ The absence of a study protocol poses a risk of selective reporting.

## 4 Discussion

TCM holds considerable therapeutic potential in the management of TBI, primarily via its potent anti-inflammatory properties that contribute to neuroprotection and functional recovery. For instance, bioactive agents derived from *Actaea racemosa* L. [Ranunculaceae; *Cimicifugae rhizoma*], *Curcuma longa* L. [Zingiberaceae; *Curcumae longae rhizoma*], and *Panax japonicus* (T.Nees) C.A.Mey. [Araliaceae; *Panacis japonici rhizoma*] have been shown to mitigate secondary neuronal injury by modulating key inflammatory signaling cascades, including NF-κB and PI3K-AKT-mTOR pathways, while downregulating pro-inflammatory cytokines such as IL-1, TNF-α, and IL-6 (Kalra et al., 2022). In parallel, *Nigella sativa* L. [Ranunculaceae; *Nigellae sativae semen*] and *Rosmarinus officinalis* L. [Lamiaceae; *Rosmarini folium*] exert antioxidative effects by enhancing endogenous antioxidant enzyme activity, thereby reducing oxidative stress and limiting neuronal apoptosis (Beheshti et al., 2016; Faridzadeh et al., 2022). Additionally, botanical drugs like *Gastrodia elata* Blume. [Orchidaceae; *Gastrodiae rhizoma*] and *C. tinctorius* L. [Asteraceae; *Carthami flos*]. have been implicated in promoting axonal regeneration and synaptic repair, which are crucial for cognitive recovery post-TBI (Kim et al., 2019; Fasina et al., 2022). Notably, TCM interventions have also demonstrated efficacy in preserving the integrity of the BBB, a key element in preventing neuroinflammatory cascades (Zheng et al., 2023). For instance, *Satureja khuzistanica* Jamzad. [Lamiaceae; *Saturejae herba*] and *Rheum officinale* Baill. [Polygonaceae; *Rhei radix et rhizoma*] significantly attenuate BBB permeability and inflammatory infiltration (Abbasloo et al., 2016; Wang et al., 2016). Furthermore, classical multi-herb formulations such as *Xuefu Zhuyu* Decoction have shown broad-spectrum neuroprotective effects, primarily through anti-inflammatory and circulation-enhancing mechanisms (Xing et al., 2016). Taken together, these findings underscore the multifaceted benefits of TCM in TBI management, with anti-inflammatory modulation as its central therapeutic axis.

Xuebijing, formulated from equal proportions of five botanical drugs—*C. tinctorius* L. [Asteraceae; *Carthami flos*], *P. lactiflora* Pall. [Paeoniaceae; *P. radix rubra*], *L. striatum* DC. [Apiaceae; *C. rhizoma*], *S. miltiorrhiza* Bunge [Lamiaceae; *Salviae miltiorrhizae radix et rhizoma*] and *A. sinensis* (Oliv.) Diels [Apiaceae; *A. sinensis radix*]—was developed using advanced extraction techniques and approved by the National Medical Products Administration in 2004 for sepsis and MODS (Wu et al., 2021; Liu et al., 2023). Our meta-analysis demonstrates that XBJ improves TBI outcomes, particularly through enhancements in GCS and GOS scores and reductions in mortality rate. Notably, the anti-inflammatory effects of XBJ, reflected in lower levels of CRP, TNF-α, and IL-6, play a central role in mitigating TBI-related inflammation. The consistent reductions in CRP, TNF-α, and IL-6 observed across studies likely reflect the attenuation of key secondary injury mechanisms following TBI. Elevated pro-inflammatory cytokines are known to trigger microglial activation, oxidative stress, and apoptotic pathways, thereby amplifying neuronal loss and BBB disruption (Beheshti et al., 2016; Faridzadeh et al., 2022). By downregulating these mediators, XBJ may interrupt the feed-forward inflammatory cascade that drives secondary neuronal injury. This pattern aligns with emerging neurotrauma biomarker evidence linking early cytokine modulation to improved long-term neurological outcomes (Kalra et al., 2022). Therefore, the observed biomarker trends in this meta-analysis may not only signify systemic anti-inflammatory activity but also suggest a broader neuroprotective role for XBJ within the context of secondary brain injury pathophysiology.

Further subgroup analyses highlight total dosage as a key factor in therapeutic efficacy. For instance, higher total dosage of XBJ were associated with greater improvements in GCS scores and reduction of inflammatory markers, with the most significant effects seen at total dosage >1,400 mL and twice-daily subgroups. These results suggest that a regimen of 100 mL per dose, administered twice daily for 2 weeks, with a total cumulative dosage >1,400 mL, may represent an optimal strategy for treating TBI. Supporting this, a well designed RCT involving 1817 sepsis patients demonstrated that administering 100 mL of XBJ twice daily for 5 days significantly reduced 28-day mortality compared to placebo (Liu et al., 2023). Additionally, the manufacturer’s instructions for XBJ recommend a single dose of 50 mL–100 mL, administered twice daily, with the frequency increasing to 3-4 times per day in severe cases. These findings underscore the dose-dependent effects of XBJ in improving clinical outcomes and reducing inflammation, emphasizing the importance of optimizing dosing regimens for maximum therapeutic efficacy.

Building on the well established anti-inflammatory benefits of XBJ, recent research has highlighted the neuroprotective and reparative properties of the certain botanical drugs of XBJ in treating central nervous system diseases. *Carthamus tinctorius* L. [Asteraceae; *Carthami flos*] reduces cerebral infarction and neurological deficits in ischemia-reperfusion injury and cerebral infarction models by mitigating free radicals and pro-inflammatory cytokines, including TNF-α and IL-1β (Fu et al., 2016; Wang Y. et al., 2020). It also addresses multiple Alzheimer’s disease mechanisms, reducing β-amyloid aggregation, inhibiting tau hyperphosphorylation, and alleviating oxidative stress, neuroinflammation, and apoptosis (Liang and Wang, 2022). *Paeonia lactiflora* Pall. [Paeoniaceae; *P. radix rubra*] confers neuroprotection in cerebral ischemia by inhibiting ferroptosis and activating autophagy via the PI3K/Akt pathway (Zhao et al., 2023). *Salvia miltiorrhiza* Bunge [Lamiaceae; *Salviae miltiorrhizae radix et rhizoma*] demonstrates protective effects in acute brain injury induced by carbon monoxide poisoning through the MAPK/ERK1/2 pathway, reducing apoptosis, promoting angiogenesis, and inhibiting ferroptosis in ischemic models (Li et al., 2022; Ko et al., 2023). *Angelica sinensis* (Oliv.) Diels [Apiaceae; *A. sinensis radix*] modulates inflammatory and autophagic pathways, inhibits NLRP3 inflammasome activation, and prevents microglial pyroptosis, offering neuroprotection in ischemic stroke (Hu et al., 2020; Luo et al., 2021; Wang et al., 2022). *Ligusticum striatum* DC. [Apiaceae; *C. rhizoma*], a major metabolite of *Da Chuanxiong* Formula, protects against BBB leakage, brain edema, and neuronal loss, enhancing neural stem cell proliferation and neuroblast differentiation, and reducing neuroinflammation in TBI models (Liu et al., 2018; Wang M. et al., 2020; Yu et al., 2021). These botanical drug of XBJ underscore its potential in mitigating neuroinflammation and supporting neural repair in TBI. However, substantial heterogeneity was observed among studies reporting CRP, TNF-α, and IL-6 outcomes. This variability may partly arise from differences in the timing of biomarker sampling, since inflammatory cytokines fluctuate dynamically during the acute and subacute phases of TBI. In addition, inconsistencies in assay methods (e.g., ELISA kits from different manufacturers), patient injury severity, and concurrent treatments (such as corticosteroids or antibiotics) could have contributed to the observed discrepancies. Although subgroup analyses based on dosage and treatment duration partly accounted for between-study variation, these residual sources of heterogeneity warrant further standardization in future clinical trials.

The safety profile of XBJ appears favorable based on available data. Among the four studies reporting ADEs, one study (Dong et al., 2017) indicated a lower incidence of ADEs in the XBJ group (7.69%) compared to the control group (10.26%), although the difference was not statistically significant (P > 0.05). Another study (Chen et al., 2017) observed mild ADEs such as diarrhea and fever in both groups, with a slightly higher incidence in the XBJ group. A third study (Hu et al., 2019) reported four cases of allergic reactions, including erythema, in the XBJ group, while a fourth study (Zhen et al., 2018) found no ADEs. Additionally, a large-scale real-world study involving 31,913 patients across 93 hospitals reported an overall ADE incidence rate of 0.30%, primarily involving mild skin lesions (Zheng et al., 2019). These results collectively suggest that XBJ administration does not significantly increase ADEs in TBI patients.

This meta-analysis offers valuable insights into XBJ for treating TBI by integrating data from various clinical studies, highlighting both its efficacy and safety. The strengths of this analysis include its comprehensive data integration, which provides a broad view of the impact of XBJ on GCS scores and inflammatory biomarkers, and the evaluation of its safety profile, indicating a generally favorable tolerance with minimal ADEs. However, several limitations must be considered. The unclear reporting of randomization and allocation concealment in several trials introduces possible selection bias, which may have led to an overestimation of XBJ’s therapeutic effects, future large-scale randomized controlled trials with transparent randomization procedures and allocation concealment are essential to strengthen the evidence base. Additionally, the predominance of studies from Mainland China involving Han Chinese populations may limit the generalizability of the findings and suggest potential regional bias. Although inflammatory biomarkers such as CRP, IL-6, and TNF-α were consistently reported across studies, neuro-specific indicators (e.g., glial fibrillary acidic protein [GFAP], ubiquitin carboxy-terminal hydrolase L1 [UCH-L1], and neuron-specific enolase [NSE]) were rarely available and thus not included in the pooled analysis. Future trials should integrate these emerging biomarkers to better elucidate XBJ’s potential neuroprotective mechanisms in TBI. The optimal dosage and regimen for XBJ remain uncertain, with our tentative recommendation of 100 mL intravenously twice daily for at least 2 weeks needing validation through larger-scale studies. Although our funnel plot did not reveal significant asymmetry, the predominance of Chinese publications suggests a risk of publication bias. Finally, the limited number of studies precludes extensive sensitivity and subgroup analyses, affecting the depth of our findings. Addressing these limitations in future research is essential to refine and validate our conclusions.

## 5 Conclusion

This meta-analysis provides evidence that XBJ is a promising adjunctive therapy for TBI. It significantly improves neurological outcomes, as reflected by higher GCS and GOS scores, and reduces mortality. The observed reductions in CRP, TNF-α, and IL-6 further support its anti-inflammatory potential. Subgroup findings indicate that higher total dosages and twice-daily intravenous administration (100 mL per dose for 2 weeks, totaling >1,400 mL) may enhance therapeutic efficacy. While these results highlight the clinical value of XBJ in TBI management, further high-quality trials are needed to confirm its benefits and optimize dosing regimens.

## Data Availability

The original contributions presented in the study are included in the article/Supplementary Material, further inquiries can be directed to the corresponding authors.
