# Supplementary material for: Therapeutic efficacy and safety of Xuebijing in traumatic brain injury: systematic review and meta-analysis
Source: Front Pharmacol. 2025 Dec 8;16:1669352. doi: 10.3389/fphar.2025.1669352 (PMC12719521; doi:10.3389/fphar.2025.1669352)
Supplement: Supplementary file 1 [file Supplementaryfile1.docx]

Supplementary Material

# Supplementary Tables

**Supplementary Table S1:** Search strategies.

| **Database** | **ID** | **Search** | **Hits** |
| --- | --- | --- | --- |
| **PubMed** | #1 | "Craniocerebral Trauma"[Mesh] Sort by: Most Recent | 187059 |
|  | #2 | Brain Injuries, Traumatic OR Brain Injuries OR Coma, Post-Head Injury OR Cranial Nerve Injuries OR Head Injuries, Closed OR Head Injuries, Penetrating OR Intracranial Hemorrhage, Traumatic OR Skull Fractures | 170823 |
|  | #3 | brain*[Title/Abstract] OR head*[Title/Abstract] OR crani*[Title/Abstract] OR craniocerebral*[Title/Abstract] OR cerebr*[Title/Abstract] OR cerebel*[Title/Abstract] OR intracran*[Title/Abstract] OR intra-cran*[Title/Abstract] OR intercran*[Title/Abstract] OR inter-cran*[Title/Abstract] OR subarachnoid*[Title/Abstract] OR skull*[Title/Abstract] OR forebrain*[Title/Abstract] OR encephalopathy[Title/Abstract] | 2577114 |
|  | #4 | injur*[Title/Abstract] OR trauma*[Title/Abstract] OR damag*[Title/Abstract] OR fracture*[Title/Abstract] OR contusion*[Title/Abstract] OR laceration*[Title/Abstract] OR concussion*[Title/Abstract] OR wound*[Title/Abstract] | 2538521 |
|  | #5 | #3 AND #4 | 383974 |
|  | #6 | #1 OR #2 OR #5 | 484530 |
|  | #7 | xuebijing[Title/Abstract] OR XBJ[Title/Abstract] | 335 |
|  | #8 | #6 AND #7 | 9 |
| **EBSCO** | ID | Search | Hits |
|  | S1 | SU Craniocerebral Trauma OR SU ( Brain Injuries, Traumatic OR Brain Injuries OR Coma, Post-Head Injury OR Cranial Nerve Injuries OR Head Injuries, Closed OR Head Injuries, Penetrating OR Intracranial Hemorrhage, Traumatic OR Skull Fractures ) | 355524 |
|  | S2 | [TI ( brain* OR head* OR crani* OR craniocerebral* OR cerebr* OR cerebel* OR intracran* OR intra-cran* OR intercran* OR inter-cran* OR subarachnoid* OR skull* OR forebrain* OR encephalopathy ) OR AB ( brain* OR head* OR crani* OR craniocerebral* OR cerebr* OR cerebel* OR intracran* OR intra-cran* OR intercran* OR inter-cran* OR subarachnoid* OR skull* OR forebrain* OR encephalopathy ) OR SU ( brain* OR head* OR crani* OR craniocerebral* OR cerebr* OR cerebel* OR intracran* OR intra-cran* OR intercran* OR inter-cran* OR subarachnoid* OR skull* OR forebrain* OR encephalopathy )](https://web.s.ebscohost.com/ehost/javascript:showHistoryTerm('ctl00_ctl00_FindField_FindField_historyControl_HistoryRepeater_ctl00_ellipsis',true)) | 11162451 |
|  | S3 | TI ( injur* OR trauma* OR damag* OR fracture* OR contusion* OR laceration* OR concussion* OR wound* ) OR AB ( injur* OR trauma* OR damag* OR fracture* OR contusion* OR laceration* OR concussion* OR wound* ) OR SU ( injur* OR trauma* OR damag* OR fracture* OR contusion* OR laceration* OR concussion* OR wound* ) | 10043835 |
|  | S4 | TI ( xuebijing OR XBJ ) OR AB ( xuebijing OR XBJ ) OR SU ( xuebijing OR XBJ ) | 858 |
|  | S5 | S2 AND S3 | 1451386 |
|  | S6 | S1 OR S5 | 1451386 |
|  | S7 | S4 AND S6 | 13 |
| **EMBASE** | ID | Search | Hits |
|  | #1 | 'head injury'/exp | 393735 |
|  | #2 | 'head injury':ab,kw,ti | 29388 |
|  | #3 | 'brain injuries, traumatic':ab,kw,ti OR 'brain injuries':ab,kw,ti OR 'coma, post-head injury':ab,kw,ti OR 'cranial nerve injuries':ab,kw,ti OR 'head injuries, closed':ab,kw,ti OR 'head injuries, penetrating':ab,kw,ti OR 'intracranial hemorrhage, traumatic':ab,kw,ti OR 'skull fractures':ab,kw,ti | 17108 |
|  | #4 | 'brain*':ab,kw,ti OR 'head*':ab,kw,ti OR 'crani*':ab,kw,ti OR 'craniocerebral*':ab,kw,ti OR 'cerebr*':ab,kw,ti OR 'cerebel*':ab,kw,ti OR 'intracran*':ab,kw,ti OR 'intra-cran*':ab,kw,ti OR 'intercran*':ab,kw,ti OR 'inter-cran*':ab,kw,ti OR 'subarachnoid*':ab,kw,ti OR 'skull*':ab,kw,ti OR 'forebrain*':ab,kw,ti OR 'encephalopathy':ab,kw,ti | 3443845 |
|  | #5 | 'injur*':ab,kw,ti OR 'trauma*':ab,kw,ti OR 'damag*':ab,kw,ti OR 'fracture*':ab,kw,ti OR 'contusion*':ab,kw,ti OR 'laceration*':ab,kw,ti OR 'concussion*':ab,kw,ti OR 'wound*':ab,kw,ti | 3298038 |
|  | #6 | 'xuebijing':ab,kw,ti OR 'xbj':ab,kw,ti | 443 |
|  | #7 | #1 OR #2 OR #3 | 396317 |
|  | #8 | #4 AND #5 | 533118 |
|  | #9 | #7 OR #8 | 722789 |
|  | #10 | #6 AND #9 | 16 |
| **Web of Science** | ID | Search | Hits |
|  | #1 | (TS=(Craniocerebral Trauma)) OR TS=(Brain Injuries, Traumatic OR Brain Injuries OR Coma, Post-Head Injury OR Cranial Nerve Injuries OR Head Injuries, Closed OR Head Injuries, Penetrating OR Intracranial Hemorrhage, Traumatic OR Skull Fractures) | 411800 |
|  | #2 | ((TS=(brain* OR head* OR crani* OR craniocerebral* OR cerebr* OR cerebel* OR intracran* OR intra-cran* OR intercran* OR inter-cran* OR subarachnoid* OR skull* OR forebrain* OR encephalopathy)) OR TI=(brain* OR head* OR crani* OR craniocerebral* OR cerebr* OR cerebel* OR intracran* OR intra-cran* OR intercran* OR inter-cran* OR subarachnoid* OR skull* OR forebrain* OR encephalopathy)) OR AB=(brain* OR head* OR crani* OR craniocerebral* OR cerebr* OR cerebel* OR intracran* OR intra-cran* OR intercran* OR inter-cran* OR subarachnoid* OR skull* OR forebrain* OR encephalopathy) | 4759268 |
|  | #3 | ((TS=(injur* OR trauma* OR damag* OR fracture* OR contusion* OR laceration* OR concussion* OR wound*)) OR TI=(injur* OR trauma* OR damag* OR fracture* OR contusion* OR laceration* OR concussion* OR wound*)) OR AB=(injur* OR trauma* OR damag* OR fracture* OR contusion* OR laceration* OR concussion* OR wound*) | 6085933 |
|  | #4 | ((TS=(xuebijing OR XBJ)) OR TI=(xuebijing OR XBJ)) OR AB=(xuebijing OR XBJ) | 813 |
|  | #5 | #2 AND #3 | 806474 |
|  | #6 | #1 OR #5 | 806474 |
|  | #7 | #4 AND #6 | 30 |
| **Cochrane** | ID | Search | Hits |
|  | #1 | MeSH descriptor: [Craniocerebral Trauma] explode all trees | 5686 |
|  | #2 | (Brain Injuries, Traumatic OR Brain Injuries OR Coma, Post-Head Injury OR Cranial Nerve Injuries OR Head Injuries, Closed OR Head Injuries, Penetrating OR Intracranial Hemorrhage, Traumatic OR Skull Fractures):ti,ab,kw | 5397 |
|  | #3 | (brain* OR head* OR crani* OR craniocerebral* OR cerebr* OR cerebel* OR intracran* OR intra-cran* OR intercran* OR inter-cran* OR subarachnoid* OR skull* OR forebrain* OR encephalopathy):ti,ab,kw | 214467 |
|  | #4 | (injur* OR trauma* OR damag* OR fracture* OR contusion* OR laceration* OR concussion* OR wound*):ti,ab,kw | 185665 |
|  | #5 | #1 OR #2 | 7351 |
|  | #6 | #3 AND #4 | 29891 |
|  | #7 | #5 OR #6 | 31036 |
|  | #8 | (xuebijing OR XBJ):ti,ab,kw | 116 |
|  | #9 | #7 AND #8 | 4 |
| **China National Knowledge Infrastructure/CNKI** | ID | Search | Hits |
|  | #1 | (SU%='颅脑'+'脑'+'头') OR (TKA='颅脑'+'脑'+'头') OR (KY='颅脑'+'脑'+'头') OR (TI='颅脑'+'脑'+'头') | 1862932 |
|  | #2 | (SU%='损伤'+'创伤'+'外伤') OR (TKA='损伤'+'创伤'+'外伤') OR (KY='损伤'+'创伤'+'外伤') OR (TI='损伤'+'创伤'+'外伤') | 1768854 |
|  | #3 | #1 AND #2 | 258544 |
|  | #4 | SU&='血必净' OR TKA='血必净' OR KY='血必净' OR TI='血必净' | 3980 |
|  | #5 | #3 AND #4 | 143 |
| **VIP's Chinese Science and Technology Journal Database/VIP** | ID | Search | Hits |
|  | #1 | (M="颅脑" OR "脑" OR "头") OR (K="颅脑" OR "脑" OR "头") OR (R="颅脑" OR "脑" OR "头") OR (T="颅脑" OR "脑" OR "头") | 1414680 |
|  | #2 | (M="损伤" OR "创伤" OR "外伤") OR (K="损伤" OR "创伤" OR "外伤") OR (R="损伤" OR "创伤" OR "外伤") OR (T="损伤" OR "创伤" OR "外伤") | 1164294 |
|  | #3 | #1 AND #2 | 185689 |
|  | #4 | M="血必净" OR K="血必净" OR R="血必净" OR T="血必净" | 3864 |
|  | #5 | #3 AND #4 | 113 |
| **Wanfang** | ID | Search | Hits |
|  | #1 | (关键词="颅脑" OR "脑" OR "头") OR (主题="颅脑" OR "脑" OR "头") OR (摘要="颅脑" OR "脑" OR "头") OR (题名="颅脑" OR "脑" OR "头") | 6345741 |
|  | #2 | (关键词="损伤" OR "创伤" OR "外伤") OR (主题="损伤" OR "创伤" OR "外伤") OR (摘要="损伤" OR "创伤" OR "外伤") OR (题名="损伤" OR "创伤" OR "外伤") | 1693923 |
|  | #3 | #1 AND #2 | 368035 |
|  | #4 | 关键词="血必净" OR 主题="血必净" OR 摘要="血必净" OR 题名="血必净" | 4567 |
|  | #5 | #3 AND #4 | 164 |
| **China Biology Medicine/CBM** | #1 | ("颅脑"[摘要:智能] OR "脑"[摘要:智能] OR "头"[摘要:智能]) OR ("颅脑"[标题:智能] OR "脑"[标题:智能] OR "头"[标题:智能]) | 1476558 |
|  | #2 | ("损伤"[摘要:智能] OR "创伤"[摘要:智能] OR "外伤"[摘要:智能]) OR ("损伤"[标题:智能] OR "创伤"[标题:智能] OR "外伤"[标题:智能]) | 954584 |
|  | #3 | #1 AND #2 | 208483 |
|  | #4 | "血必净"[标题:智能] OR "血必净"[摘要:智能] | 3614 |
|  | #5 | #3 AND #4 | 120 |
| **Last Run Date: 08/02/2025** | | | |

##
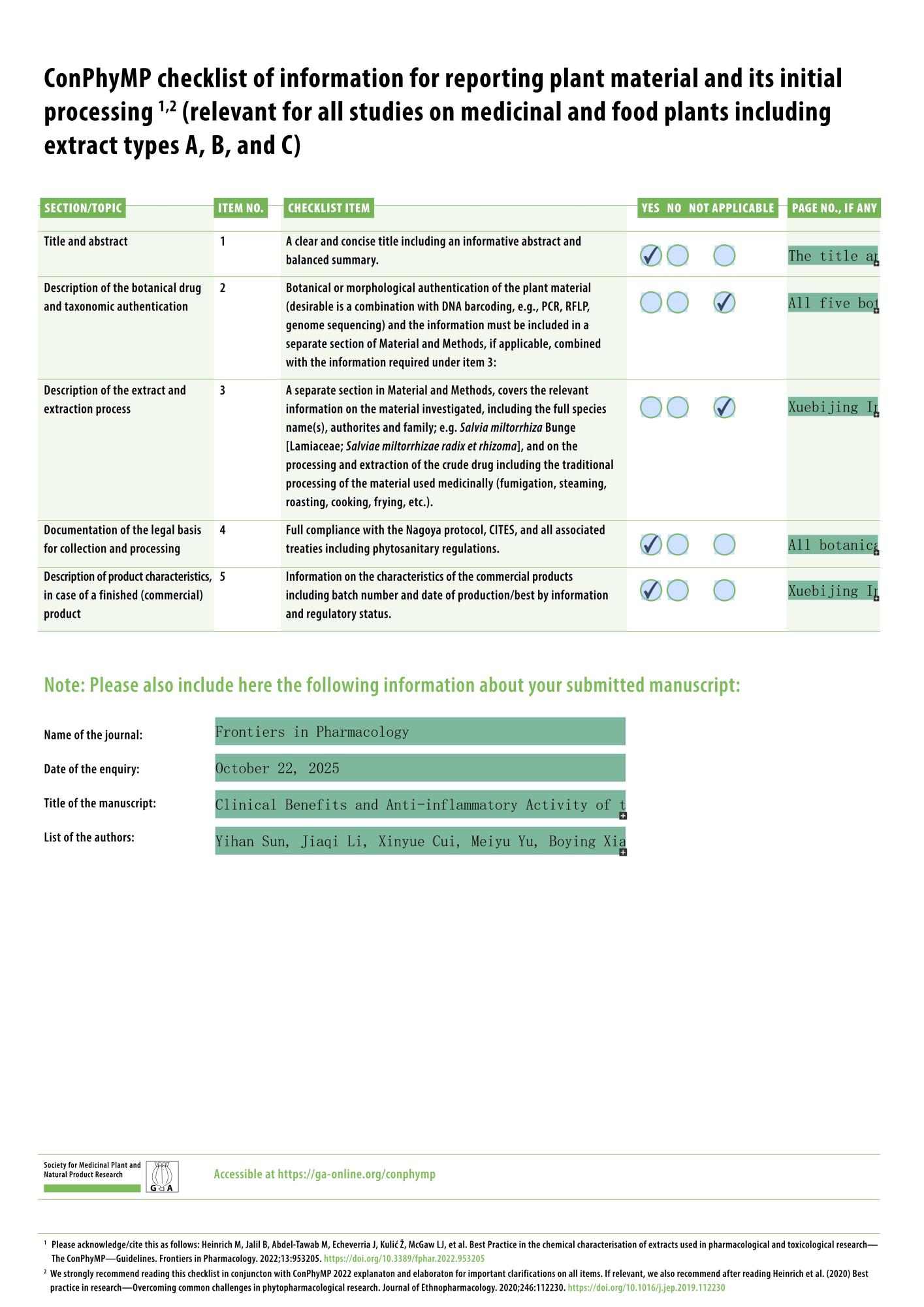
Supplementary Table S2: ConPhyMP-checklists.


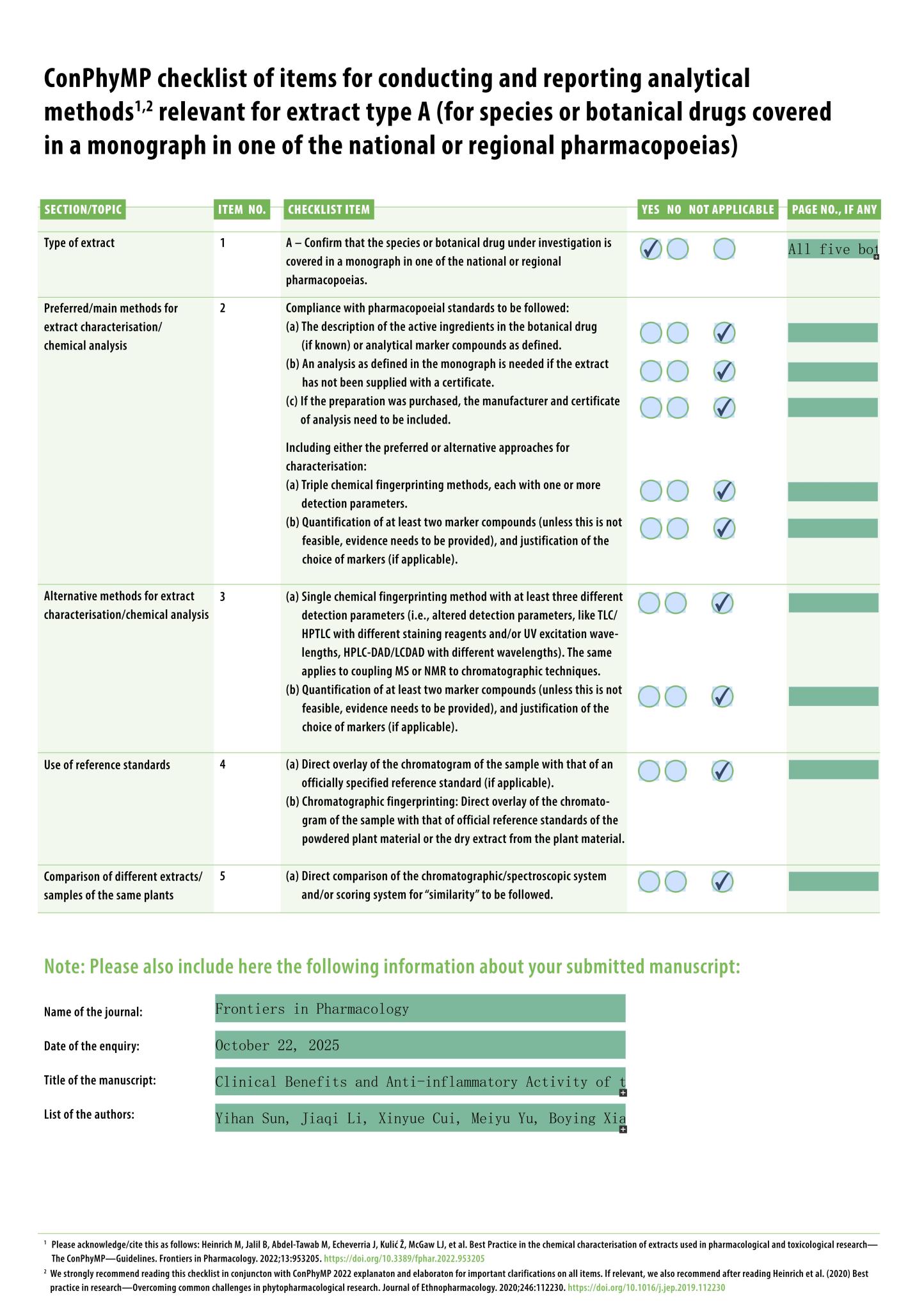


# Supplementary Figures


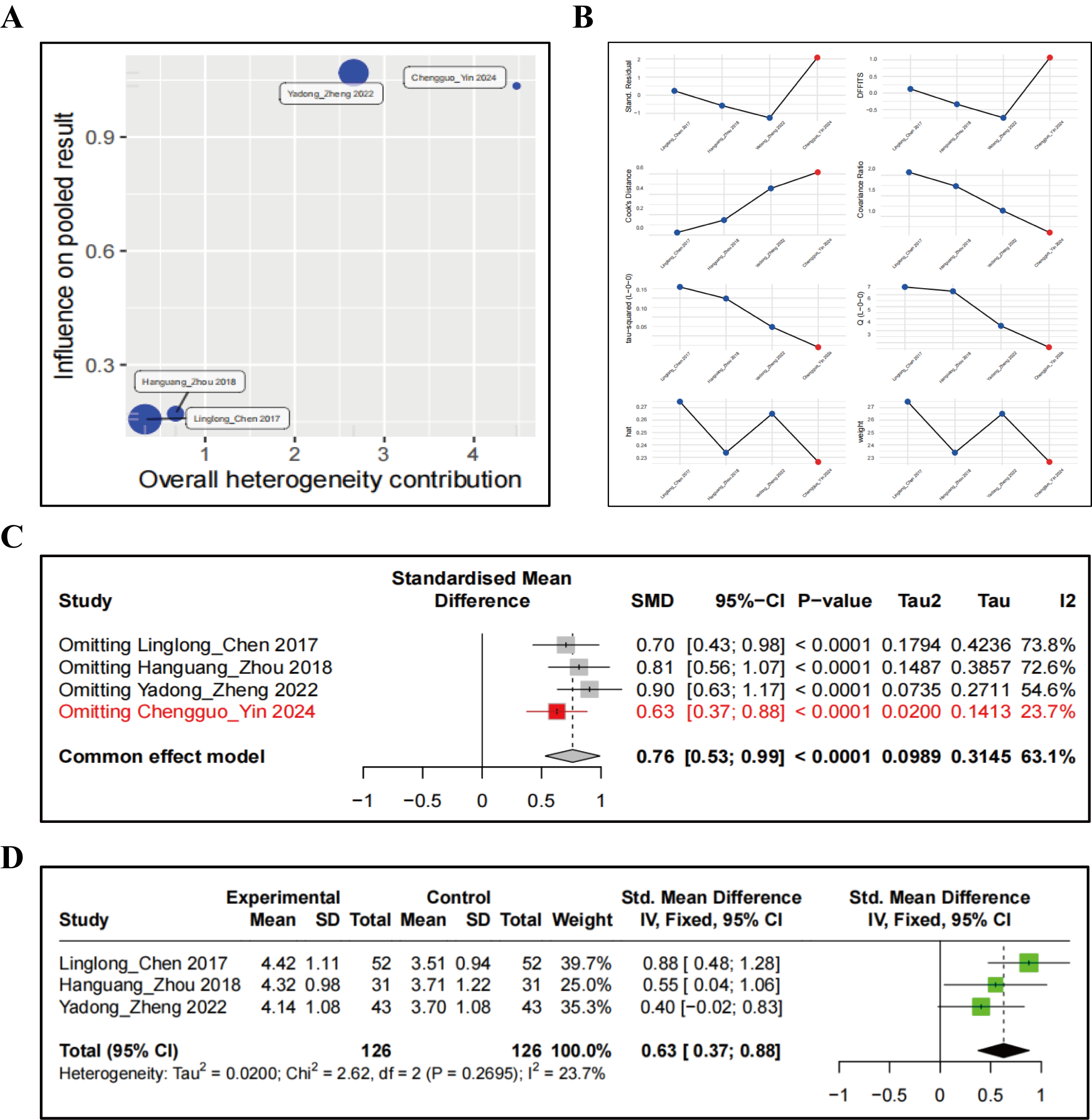


**Supplementary Figure S1.** Sensitivity analysis of GOS. (A) The Baujat plot, (B) influence diagnostic analysis and (C) leave-one-out sensitivity analysis identified the study by Chengguo Yin (2024) as outlier, indicating its significant contribution to overall heterogeneity and influence on the pooled effect. (D) Forest plot showing the effect of XBJ compared to the control group on GOS after exclusion of the outlier study.

**
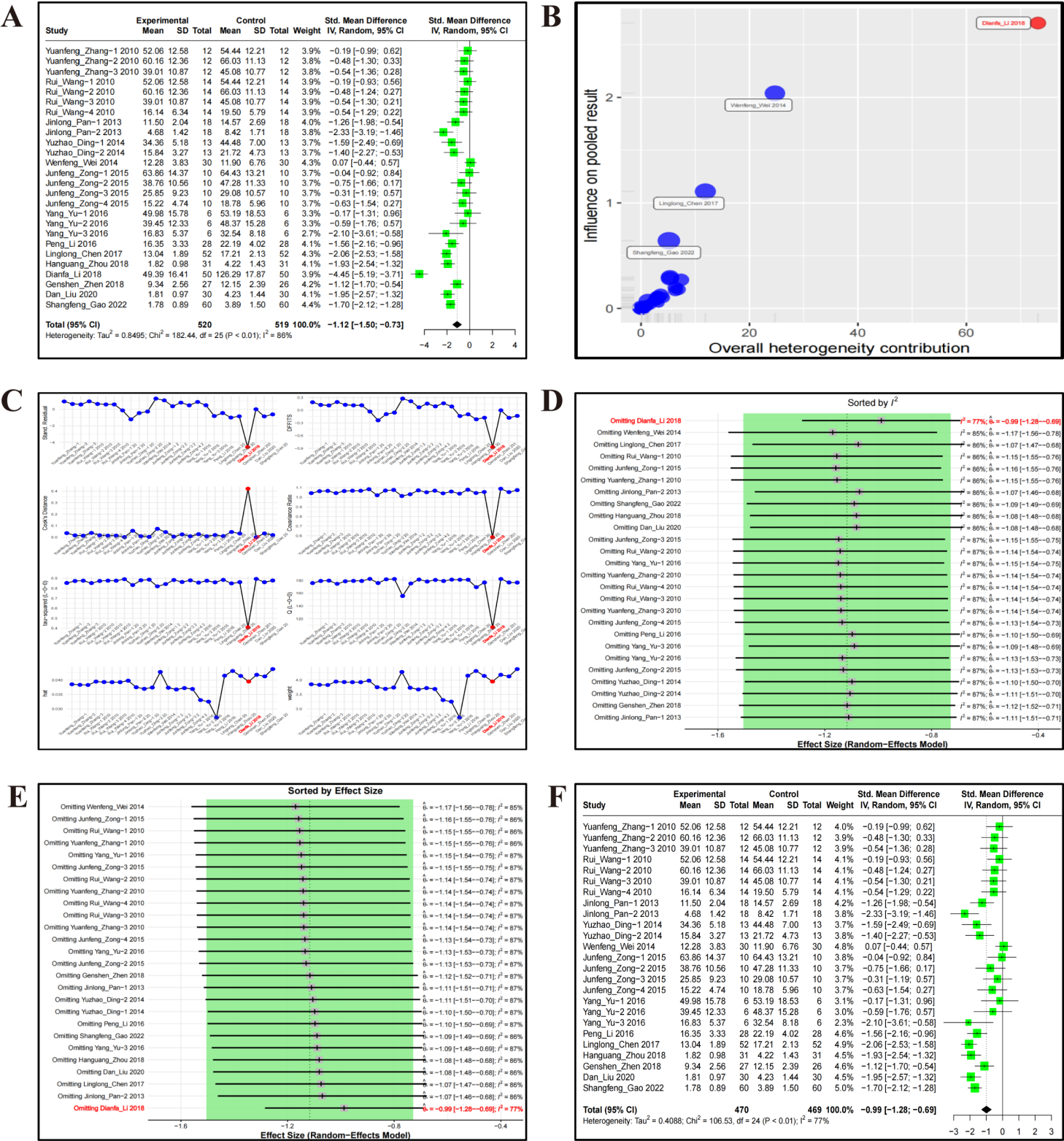
**

**Supplementary Figure S2.** Sensitivity analysis of CRP. (A) Forest plot illustrating the effects of XBJ compared to the control group on CRP levels with significant heterogeneity. (B) The Baujat plot, (C) influence diagnostic analysis and (D, E) leave-one-out sensitivity analysis identified the study by Dianfa Li (2018) as outlier, indicating its significant contribution to overall heterogeneity and influence on the pooled effect. (F) Forest plot showing the effect of XBJ compared to the control group on CRP after exclusion of the outlier study.


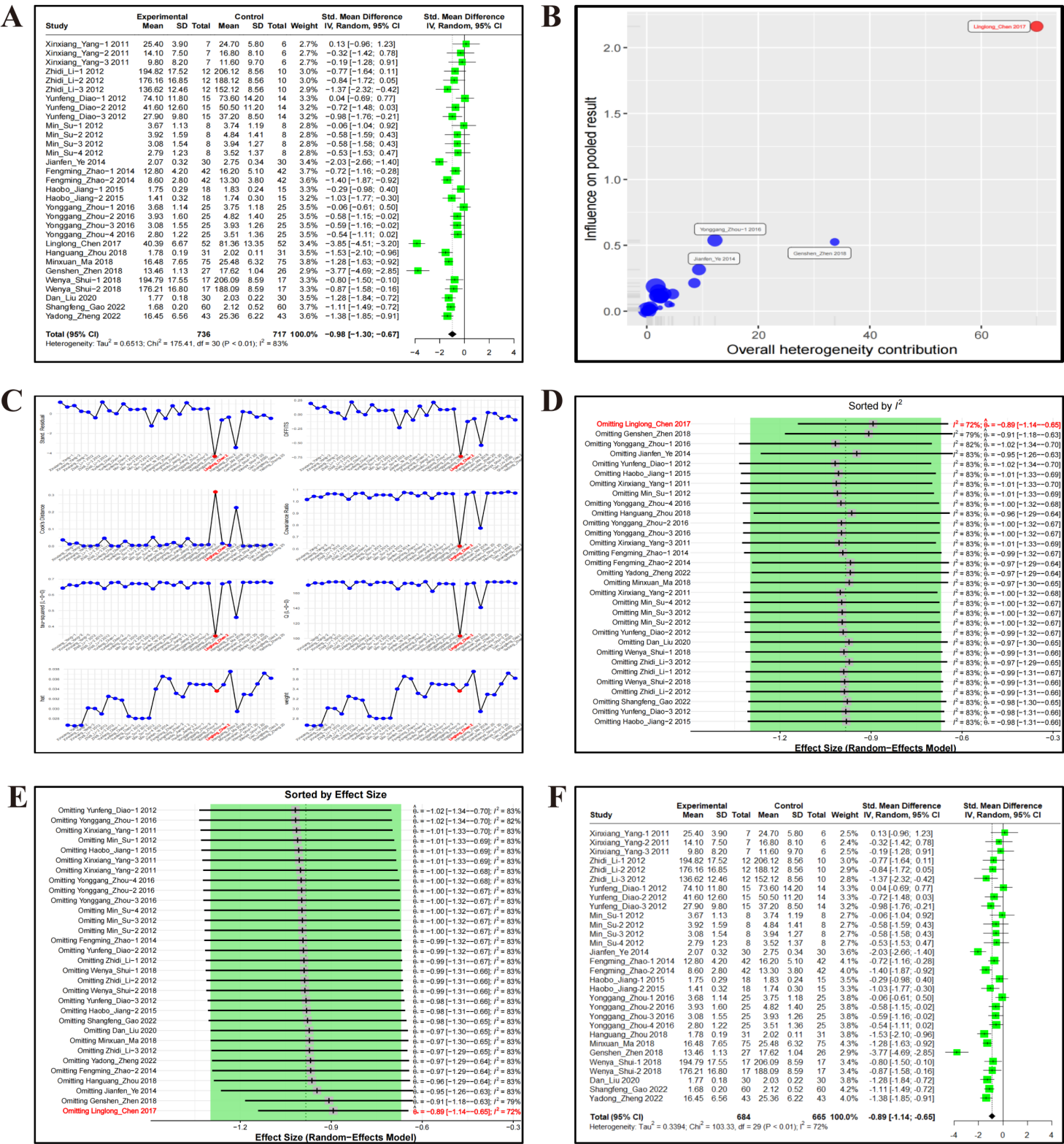


**Supplementary Figure S3.** (A) Forest plot illustrating the effects of XBJ compared to the control group on TNF-α levels with significant heterogeneity. (B) The Baujat plot, (C) influence diagnostic analysis and (D, E) leave-one-out sensitivity analysis identified the study by Lingrong Chen, 2017 as outlier, indicating its significant contribution to overall heterogeneity and influence on the pooled effect. (F) Forest plot showing the effect of XBJ compared to the control group on TNF-α after exclusion of the outlier study.


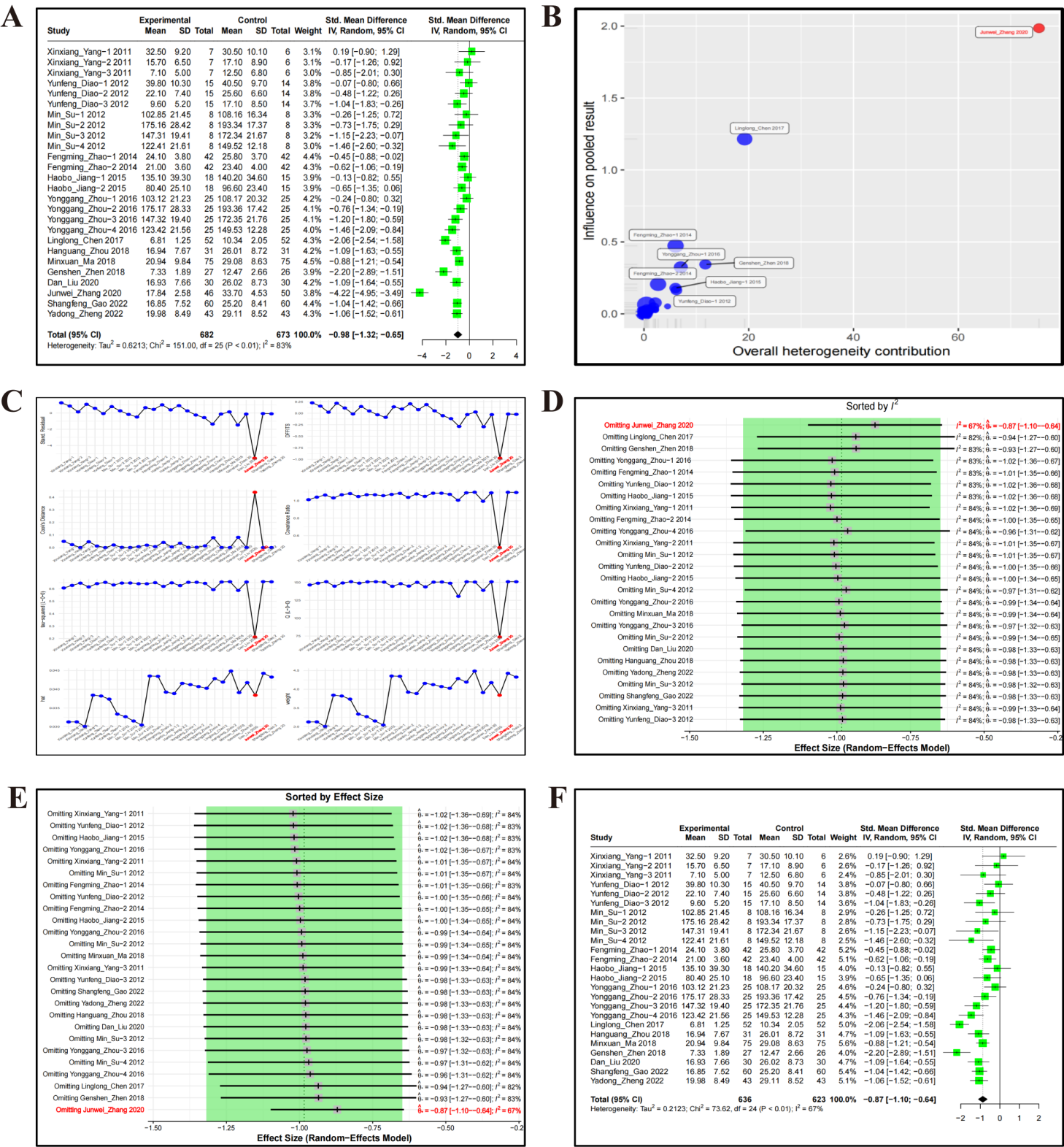


**Supplementary Figure S4.** (A) Forest plot illustrating the effects of XBJ compared to the control group on IL-6 levels with significant heterogeneity. (B) The Baujat plot, (C) influence diagnostic analysis and (D, E) leave-one-out sensitivity analysis identified the study by Junwei Zhang, 2020 as outlier, indicating its significant contribution to overall heterogeneity and influence on the pooled effect. (F) Forest plot showing the effect of XBJ compared to the control group on IL-6 after exclusion of the outlier study.


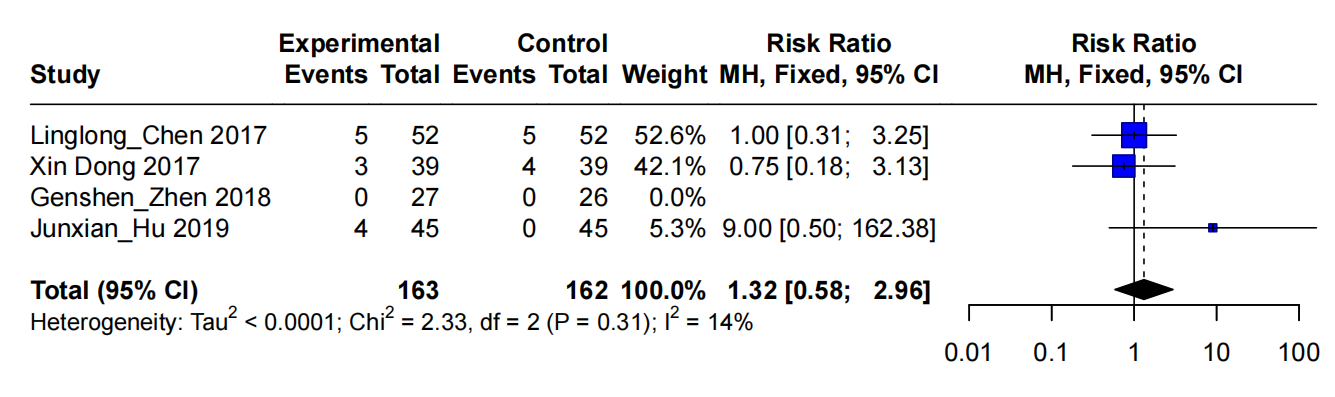


**Supplementary Figure S5：**Forest plot illustrates the effects of XBJ compared to the control group on ADE. ADE: Adverse Drug Event.

**
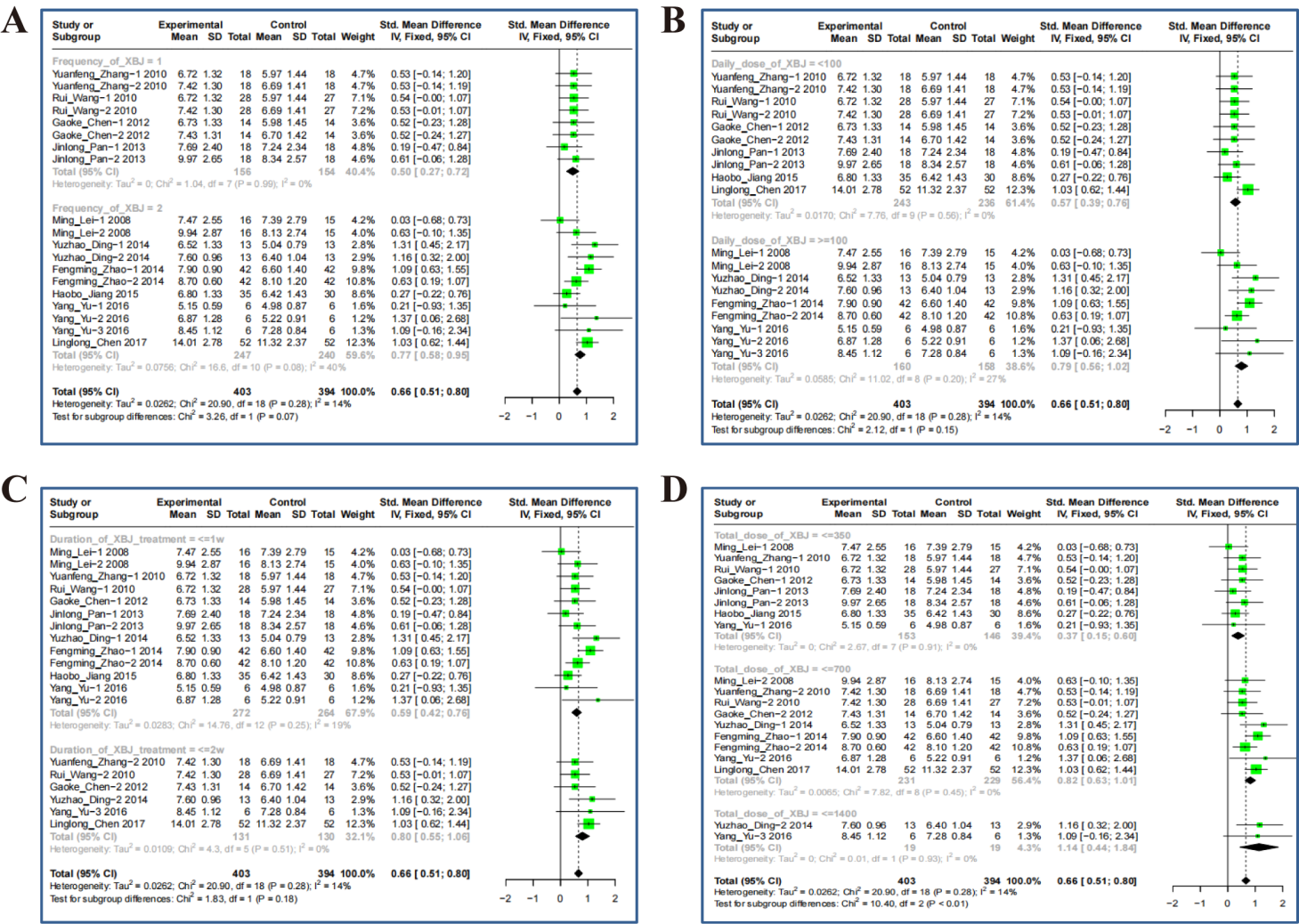
**

**Supplementary Figure S6：**Subgroup analysis of GCS based on (A) frequency, (B) daily dosage, (C) treatment duration and (D) total dosage.

**
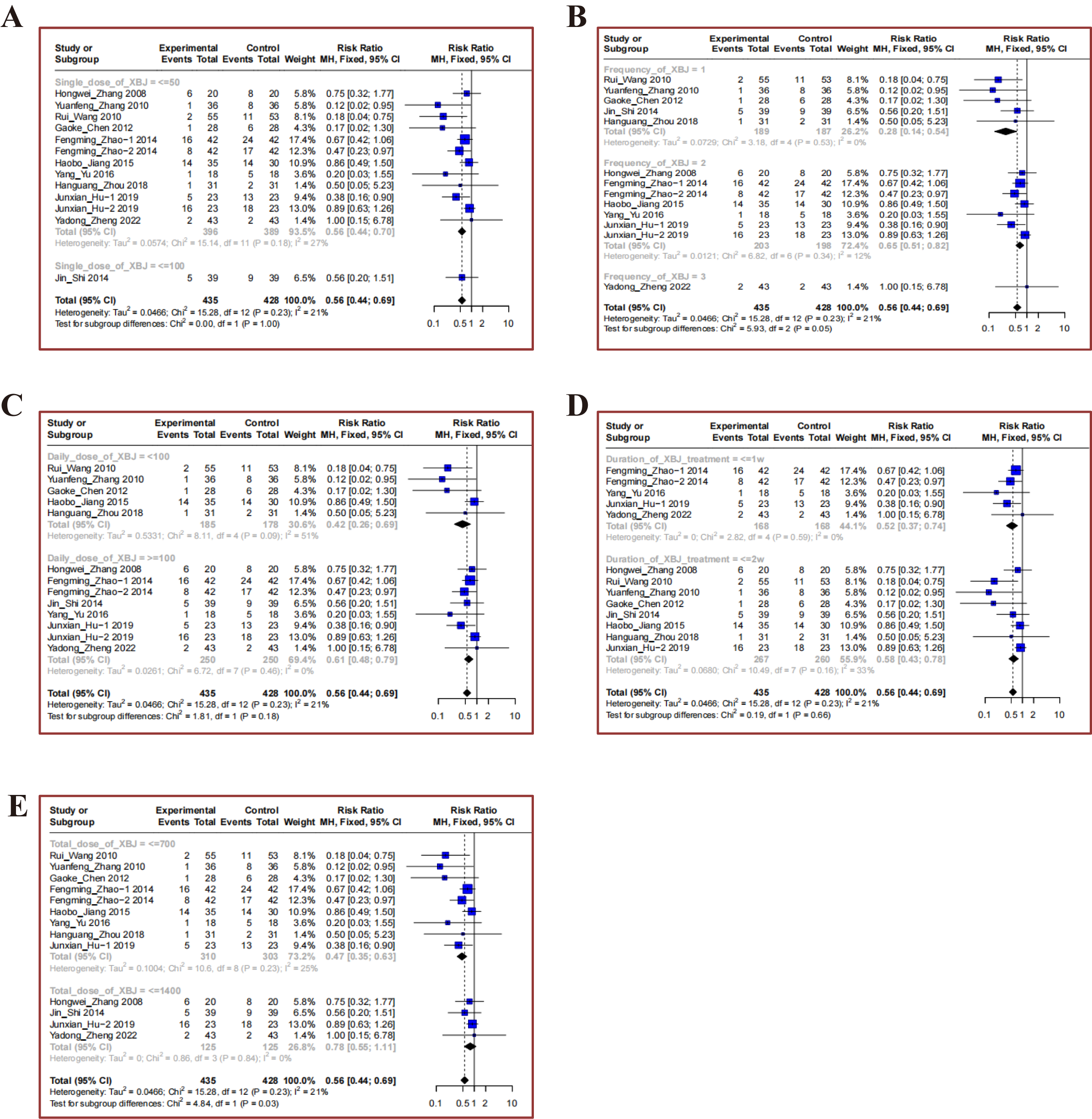
**

**Supplementary Figure S7：**Subgroup analysis of mortality based on (A) single dose, (B) frequency, (C) daily dosage, (D) treatment duration and (E) total dosage.


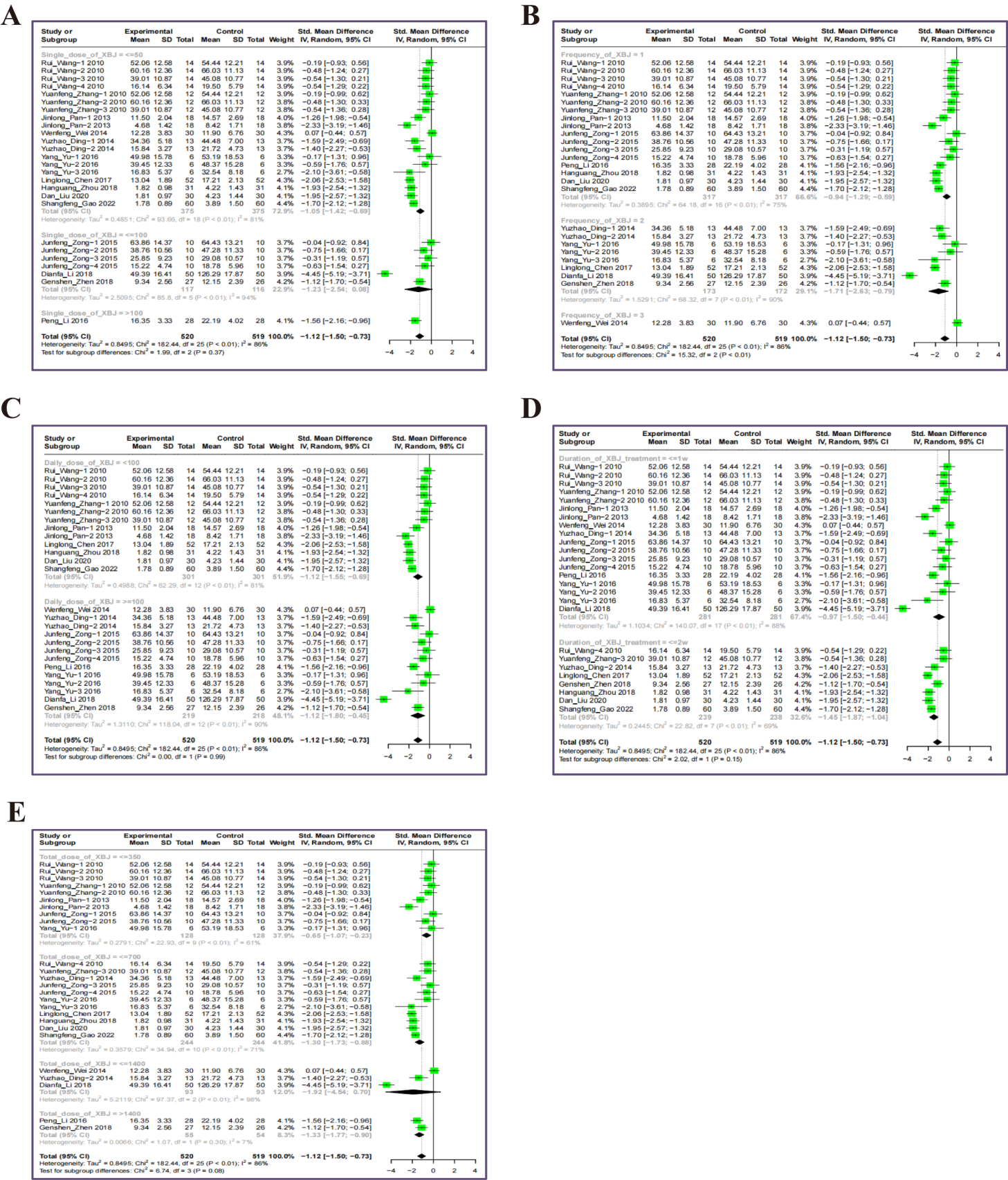


**Supplementary Figure S8**：Subgroup analysis of CRP based on (A) single dose, (B) frequency, (C) daily dosage, (D) treatment duration and (E) total dosage.

**
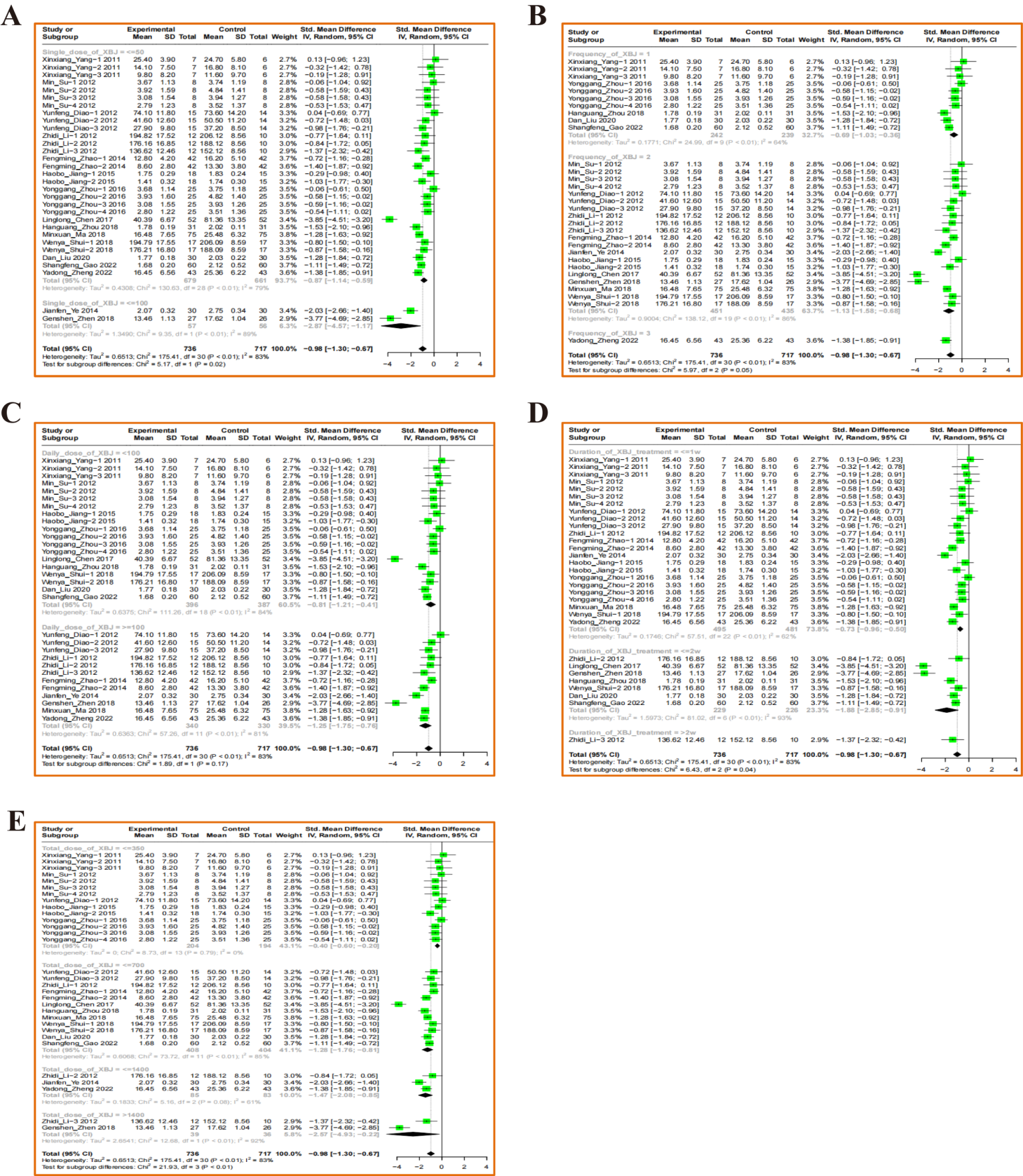
**

**Supplementary Figure S9**：Subgroup analysis of TNF-α based on (A) single dose, (B) frequency, (C) daily dosage, (D) treatment duration and (E) total dosage.

**
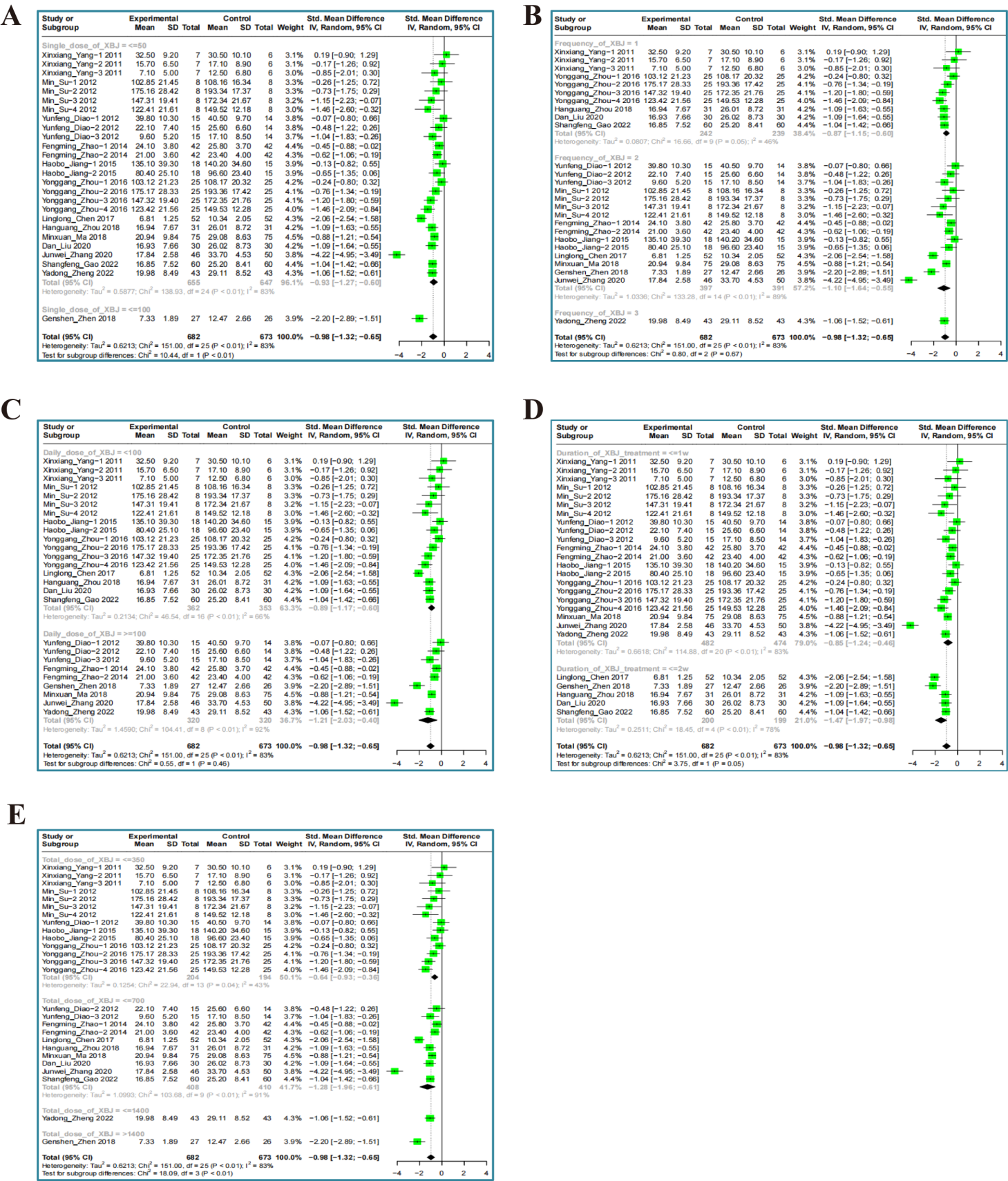
**

**Supplementary Figure S10：**Subgroup analysis of IL-6 based on (A) single dose, (B) frequency, (C) daily dosage, (D) treatment duration and (E) total dosage.
